# Cognitive Effects Following Offline High-Frequency Repetitive Transcranial Magnetic Stimulation (HF-rTMS) in Healthy Populations: A Systematic Review and Meta-Analysis

**DOI:** 10.1007/s11065-023-09580-9

**Published:** 2023-03-01

**Authors:** Mei Xu, Stevan Nikolin, Nisal Samaratunga, Esther Jia Hui Chow, Colleen K. Loo, Donel M. Martin

**Affiliations:** 1 Discipline of Psychiatry & Mental Health, School of Clinical Medicine, Faculty of Medicine, University of New South Wales, Sydney, Australia; 2 Black Dog Institute, Sydney, Australia; 3 The George Institute for Global Health, Sydney, Australia; 4 UNSW Sydney, High St, Kensington, NSW 2052 Australia

**Keywords:** Offline high-frequency repetitive transcranial magnetic stimulation, Offline HF-rTMS, Cognition, Meta-analysis

## Abstract

High-frequency repetitive transcranial magnetic stimulation (HF-rTMS) is a commonly used form of rTMS to treat neuropsychiatric disorders. Emerging evidence suggests that ‘offline’ HF-rTMS may have cognitive enhancing effects, although the magnitude and moderators of these effects remain unclear. We conducted a systematic review and meta-analysis to clarify the cognitive effects of offline HF-rTMS in healthy individuals. A literature search for randomised controlled trials with cognitive outcomes for pre and post offline HF-rTMS was performed across five databases up until March 2022. This study was registered on the PROSPERO international prospective protocol for systematic reviews (PROSPERO 2020 CRD 42,020,191,269). The Risk of Bias 2 tool was used to assess the risk of bias in randomised trials. Separate analyses examined the cognitive effects of excitatory and inhibitory forms of offline HF-rTMS on accuracy and reaction times across six cognitive domains. Fifty-three studies (N = 1507) met inclusion criteria. Excitatory offline HF-rTMS showed significant small sized effects for improving accuracy (k = 46, g = 0.12) and reaction time (k = 44, g = -0.13) across all cognitive domains collapsed. Excitatory offline HF-rTMS demonstrated a relatively greater effect for executive functioning in accuracy (k = 24, g = 0.14). Reaction times were also improved for the executive function (k = 21, g = -0.11) and motor (k = 3, g = -0.22) domains following excitatory offline HF-rTMS. The current review was restricted to healthy individuals and future research is required to examine cognitive enhancement from offline HF-rTMS in clinical cohorts.

**Supplementary Information:**

The online version contains supplementary material available at 10.1007/s11065-023-09580-9.

## Introduction

Transcranial magnetic stimulation (TMS) is a non-invasive brain stimulation technique that has become an established investigative and therapeutic tool in neurology, psychiatry, and cognitive neuroscience. It involves the application of strong focal magnetic fields to modulate brain activity via the principal of electromagnetic induction. Repetitive TMS (rTMS), which involves the delivery of multiple pulses of stimulation in close succession, has been shown to generate long-lasting, cumulative after-effects on brain function (Rotenberg et al., 2014). Correspondingly, rTMS is commonly used as a research and therapeutic tool (Guo et al., 2017). Of potential clinical significance, sham-controlled trials have shown modest cognitive enhancing effects following a treatment course of rTMS in people with depression (Martin et al., 2017), mild cognitive impairment (Marra et al., 2015) and Alzheimer’s disease (Lee et al., 2016; Sabbagh et al., 2019). These traditional protocols have typically used high-frequency rTMS (HF-rTMS), as this form of rTMS is thought to produce relatively promising excitatory neuromodulatory effects compared to low-frequency rTMS (LF-rTMS) (Fitzgerald & Daskalakis, 2013; Rotenberg et al., 2014). Despite these promising results, it remains unclear which aspects of cognition (i.e., accuracy, reaction time) are most affected, or which HF-rTMS protocols are optimal for producing cognitive enhancing effects.

rTMS can induce facilitatory (i.e., enhance brain functioning) or inhibitory effects (i.e., disrupt brain functioning) that outlast the stimulation duration for minutes to hours or even days (Lefaucheur et al., 2014). These excitatory or inhibitory aftereffects are largely dependent upon stimulation parameters, particularly pulse frequency (Beynel et al., 2019; Walsh & Cowey, 2000). Generally, rTMS protocols include low-frequency rTMS (LF-rTMS, < = 1 Hz) or HF-rTMS (> = 5 Hz) (Dell'Osso & Di Lorenzo, 2020; Fitzgerald et al., 2006; Hallett, 2007; Luber & Lisanby, 2014). There is a consensus that LF-rTMS tends to be inhibitory, inducing long-term depression (LTD)-like plasticity. Conversely, HF-rTMS is considered excitatory, producing long-term potentiation (LTP)-like neuroplasticity (Fitzgerald & Daskalakis, 2013; Rotenberg et al., 2014; Walsh & Cowey, 2000). Notwithstanding, this is not always the case. RTMS administered over the motor cortex has demonstrated increased cortical excitability following LF-rTMS (Fitzgerald et al., 2006; Gilio et al., 2003), as well as no excitatory changes after HF-rTMS (Fitzgerald et al., 2006; Lazzaro et al., 2002), indicating that overall neuromodulatory effects can also vary between studies or samples.

A more recent addition to HF-rTMS protocols is theta-burst stimulation (TBS) (Dell'Osso & Di Lorenzo, 2020; Huang et al., 2005). TBS involves 50 Hz bursts delivered at a theta frequency of 5 Hz, which mimics the neuronal firing patterns of the brain (Huang et al., 2005). Intermittent theta-burst stimulation (iTBS) has been shown to facilitate cortical plasticity (Huang et al., 2005; Rotenberg et al., 2014). Recently, iTBS was approved by the US Food and Drug Administration (FDA) for the treatment of refractory depression (Blumberger et al., 2018; Chu et al., 2021; Li et al., 2014) and is increasingly being used in clinical research. Conversely, continuous theta-burst stimulation (cTBS) produces synaptic suppression and decreases cortical excitability (Huang et al., 2005; Rotenberg et al., 2014). Although TBS is different with regards to the patterned form of stimulation (i.e., combining HF-rTMS at different frequencies: theta and gamma) which has differing neuromodulatory effects for iTBS and cTBS (i.e., excitatory and inhibitory), it still involves high frequency stimulation as per current accepted definitions. Recent studies have suggested that TBS holds promise for enhancement in some cognitive domains (e.g., memory, executive functioning) (Hoy et al., 2016; Lowe et al., 2018; Tambini et al., 2017).

In addition to different neuromodulatory effects that vary according to stimulation frequency, the cognitive effects of HF-rTMS can also vary depending on the timing of administration. To modulate cognition, HF-rTMS can be administered either ‘online’ or ‘offline’. For ‘online’ protocols, participants perform a task or activity during HF-TMS. The effects of ‘online’ stimulation are generally short-lasting, less than a few seconds (Miniussi et al., 2008), inducing a temporary disruption or facilitation of ongoing processing. In contrast, ‘offline’ protocols involve a continuous session or repeated treatments of HF-rTMS delivered at rest, immediately followed by a cognitive task. The cognitive effects of ‘offline’ HF-rTMS are therefore related to neurophysiological changes that outlast the period of stimulation. Proposed mechanisms for these effects have included: direct targeted cortical facilitation that results in more effective processing, changes in neural oscillatory activity, synaptic changes, and addition-by-subtraction in which TMS might produce cognitive enhancement via disruption of competing cognitive processing (Luber & Lisanby, 2014). TMS studies coupling with other neuroimaging techniques (e.g., magnetic resonance spectroscopy (MRS), electroencephalogram (EEG), magnetic resonance imaging (MRI)) has provided some insights into potential biological correlates of offline HF-rTMS cognitive effects, identifying changes in cortical excitability and neurotransmitter activity (Allen et al., 2014), electrophysiology (Barr et al., 2009; Chung et al., 2018) and functional connectivity (Wang & Voss, 2015; Wang et al., 2014).

The modulating effects of offline HF-rTMS on cognition are likely associated with spatial parameters of the stimulation (i.e., stimulation sites, targeting methods, stimulation intensity, coil geometry and spatial alignment relative to cortical neurons) and temporal parameters (i.e., stimulation pulse frequency, the number of sessions, pulses per session, waveform, inter-trial interval) (Beynel et al., 2019; Rotenberg et al., 2014). Stimulation pulse frequency, in particular, may be important. A recent meta-analysis of ‘online’ TMS cognitive effects found that higher frequencies (i.e., 10 Hz and 20 Hz) were associated with larger negative cognitive effects (Beynel et al., 2019). The cognitive effects of offline HF-rTMS may also be moderated by the cumulative benefits of repeated sessions of stimulation. For example, multiple sessions of HF-rTMS produced larger cognitive effects relative to a single session in participants with Parkinson’s disease (Jiang et al., 2020). In addition, the targeting method for rTMS has been identified as an important factor, particularly for the treatment of neuropsychiatric conditions (Cash et al., 2020). Commonly used targeting methods have included scalp measurement relative to the motor hot spot, the 10–20 EEG system, MRI-guided and fMRI-guided neuronavigation (Sack et al., 2009). Recent evidence suggests that neuronavigated approaches may result in relatively larger cognitive effects (Beynel et al., 2019). The reported effects of offline HF-rTMS on cognition could additionally depend on the sham or control condition used (e.g., active control site, use of coil angular rotation (45°, 90°), or sham coil). These control conditions differ in relation to induced somatosensory effects (Duecker & Sack, 2015) and the potential for neuromodulatory effects (Lisanby et al., 2001; Loo et al., 2000), which may in turn compromise blinding and moderate cognitive effects.

This study aimed to clarify the cognitive effects of offline HF-rTMS in healthy subjects and investigate the role of stimulation parameters and methodological factors in modulating cognitive outcomes. Specifically, we examined the effects of conventionally defined excitatory offline HF-rTMS (standard HF-rTMS and iTBS) protocols and inhibitory offline HF-rTMS paradigm (cTBS) on accuracy and reaction time across a range of key cognitive domains, namely attention, memory, motor, perception, language, and executive function. Secondary exploratory analyses examined the moderating effects of stimulation pulse frequency, the number of sessions, targeting method and control condition on reported cognitive effects.

## Methods

We performed a systematic review and meta-analysis in accordance with the recommendations of the Cochrane Handbook for Systematic Reviews of Interventions (Higgins et al., 2019), and PRISMA guidelines (Liberati et al. 2009). The search protocol for this study was registered on the PROSPERO international prospective protocol for systematic reviews (PROSPERO 2020 CRD 42,020,191,269, https://www.crd.york.ac.uk/PROSPERO/display_record.php?RecordID=191269).

### Literature Search

Three authors (NS, DM and MX) conducted a literature search in the following databases: PubMed, MEDLINE, Embase, Cochrane Library and PsychINFO from the first date available to 26 March 2022. Inclusion criteria were: (1) participants: healthy participants without history of psychiatric or neurologic disorders; (2) intervention: offline HF-rTMS delivered at frequencies equal to or greater than 5 Hz; (3) comparison: sham-controlled or active control sites trials; (4) outcomes: accuracy and reaction time of cognitive tasks performed before and after HF-rTMS; (5) studies: randomised controlled trials with parallel design (subjects are assigned to different stimulation conditions) or cross-over design (subjects receive a sequence of different stimulation conditions), no case studies (see Table 1). All human, English-language studies were included. Further, we searched Google Advanced Search, Pandora, Grey Matters: a practical search tool for evidence-based medicine and New York Academy of Medicine: the grey literature report for grey literature but did not identify additional relevant studies.

**Table 1:** Inclusion and exclusion criteria

Study Characteristics	Inclusion Criteria	Exclusion Criteria
Population	Healthy adults Age > = 18 Males or females	History of psychiatric or neurologic illness
Interventions	Offline TMS Any coil Targeting any brain region	Low frequency rTMS (< 5 Hz) Online TMS Single/paired pulse TMS
Comparators	Active control site or sham rTMS	No control or sham
Outcomes	Reaction time and accuracy on standardized cognitive tasks reported for pre and post rTMS (Means and SDs)	Data reported only for post rTMS
Timing	Tasks performed pre and post rTMS	Tasks performed during the stimulation
Study design	Randomised controlled trials (parallel or cross-over)	Case study Uncontrolled study

The search terms were: “cognitive task” OR “cognitive process” OR “cognition” OR “cognitive” OR “memory” OR “working memory” OR “visual memory” OR “verbal memory” OR “attention” OR “learning” OR “visual task” OR “vision” OR “visuospatial ability” OR “processing speed” OR “language” OR “decision making” OR “decision-making” OR “perception” OR “reasoning” OR “executive function” OR “cognitive function” OR “global cognitive function” AND ‘‘offline high frequency repetitive transcranial magnetic stimulation’’ OR ‘‘offline high frequency rTMS’’ OR “high frequency TMS” OR “high-frequency TMS” OR “HF-rTMS” OR “offline TMS” OR “transcranial magnetic stimulation” OR “TMS” OR “repetitive transcranial magnetic stimulation” OR “rTMS” OR “theta burst stimulation” OR “TBS”. Seven additional studies investigating the cognitive effects of offline HF-rTMS were included. Four studies came from the reference list of a recent meta-analysis study (Patel et al., 2020), two studies were sourced from the reference list of included studies (Zhang & Fong, 2019), and one study was cited in a systematic review (Martin et al., 2016).

### Study Selection

**Fig. 1:**
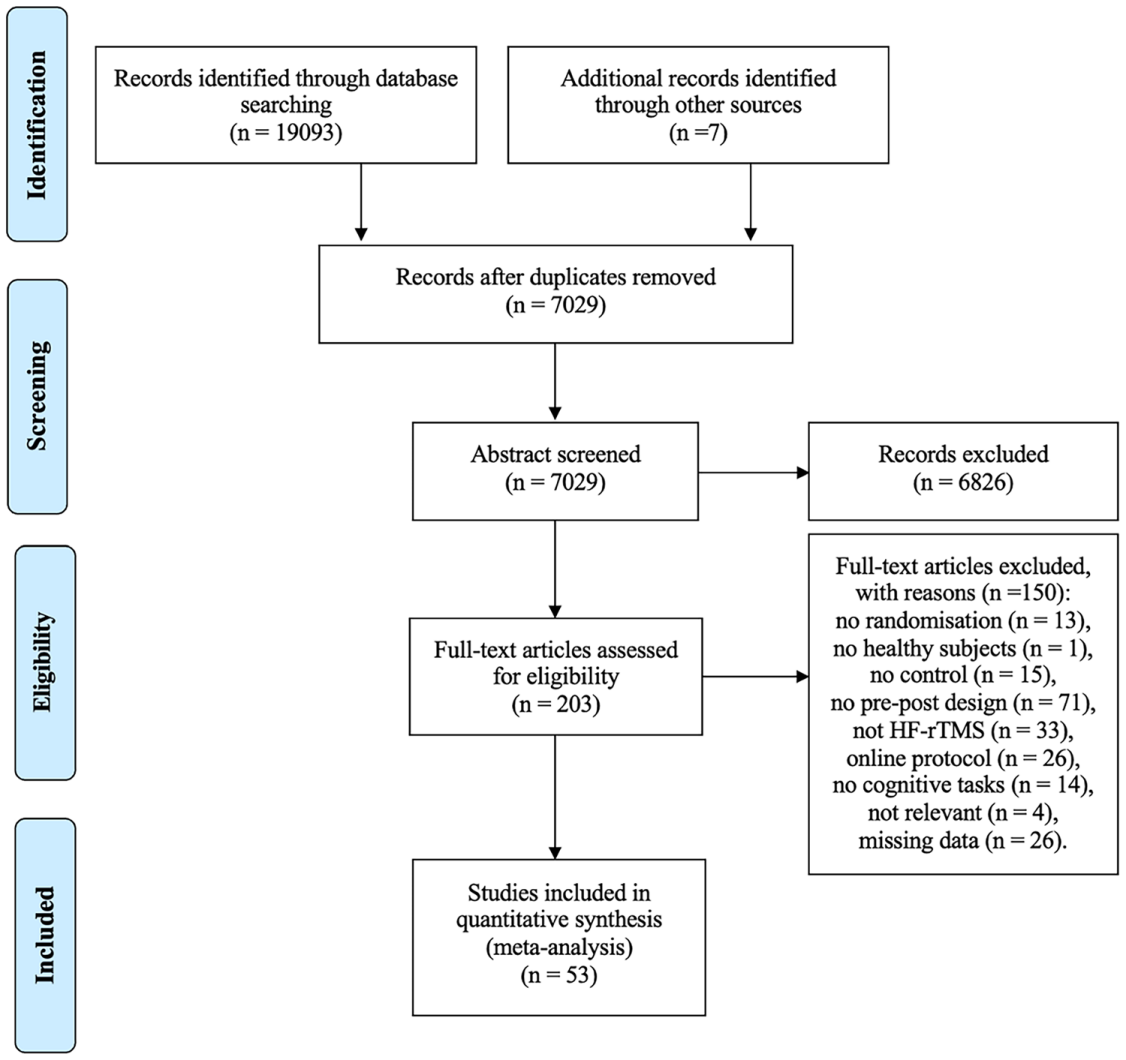
Flow diagram of study search and selection process. *Note:* In some cases, full text excluded studies met more than two exclusion criteria. Therefore, the total number of all excluded studies with reasons is more than 150

All review articles, conference abstracts and duplicates were removed. Studies were required to meet the inclusion criteria and exclusion criteria listed above. Two authors (MX and NS) independently screened the titles, abstracts, and full-text articles identified during the systematic review (see Fig. 1). Disagreements over the eligibility of particular studies were resolved through group discussion with the principal investigator (DM).

### Risk of bias Assessment

Two authors (MX and EC) independently used a revised tool to assess the risk of bias in randomised trials (RoB 2) (Sterne et al., 2019). All included studies were assessed in the following domains: (1) bias arising from the randomisation process; (2) bias due to deviations from intended interventions; (3) bias due to missing outcome data; (4) bias in measurement of the outcome; (5) bias in selection of the reported result. The possible risk-of-bias judgements include ‘low risk of bias’, ‘some concerns’ and ‘high risk of bias’ for each domain. Any discrepancies were resolved through group discussion with the principal investigator (DM).

### Data Extraction

Two authors (MX and NS) extracted the cognitive data and the following additional study information: 1) the sample sizes of active rTMS groups and control rTMS groups; 2) participant demographics (i.e., age, gender); 3) study design and treatment parameters (i.e., site of stimulation, targeting method, stimulation pulse frequency, intensity, coil type, the number of sessions, the number of pulses per session, and control method); 4) specific cognitive tasks and cognitive domains; 5) the means and standard deviations (SD) or standard errors of the mean (SEM) of the cognitive measurements before and after rTMS. Where necessary, data was extracted from figures using “WebPlotDigitizer 4.4” (Rohatgi, 2020). Corresponding authors of included studies were contacted for unreported data or additional details.

### Quantitative Synthesis

We extracted pre- and post-rTMS cognitive assessments or change scores for active and control conditions in identified studies. As it was possible that reaction times may be more sensitive for detecting cognitive effects of HF-rTMS due to the potential for ceiling effects for accuracy in healthy participants, we extracted the accuracy and reaction times separately as outcome measures from the cognitive assessments. This similar approach has also taken in a recent online rTMS meta-analyses (Beynel et al., 2019). Cognitive tasks were categorised into six cognitive domains (Martin et al., 2016; Patel et al., 2020) consisting of attention, memory, motor, language, perception, and executive functioning including updating ability, shifting ability and inhibition ability (Miyake & Friedman, 2012) (see Supplementary Materials Table S2). For those studies which provided pre- and post-stimulation cognitive outcomes, we computed effect sizes based on change scores from pre-rTMS for both active and control conditions (Higgins et al., 2019). For both parallel and crossover studies, we conservatively imputed the standard deviation of change scores (see Supplementary Materials Table S1 Eq. 1 and 2) for active and control groups by assuming a correlation coefficient of ${r}_{pre\&post}=0.5$ between pre- and post-stimulation behavioural outcomes (Pearson & Smart, 2018). If only SEMs of outcome measures at pre- and post-stimulation were reported, SEMs were converted to SDs ($SD=SEM \times \sqrt{N}$, N: sample size).

For accuracy and reaction time datasets, effect sizes were calculated as the standardised mean difference (SMD) (see Supplementary Materials Table S1 Eq. 3 and 4) using Hedge's g (see Supplementary Materials Table S1 Eq. 7 and 8) (Hedges, 1982; Broenstein et al., 2021; Higgins et al., 2019). For cross-over studies, SMD were adjusted by using a correlation coefficient of ${r}_{active\&control}=0$ (Pearson & Smart, 2018; Sloan et al., 2020) between conditions (see Supplementary Materials Table S1 Eq. 5 and 6) with corrected Hedges’ g (see Supplementary Materials Table S1 Eq. 9 and 10) (Gibbons et al., 1993; Broenstein et al., 2021). The above correlation coefficients (${r}_{pre\&post}=0.5, {r}_{active\&control}=0$) were found to be conservative estimates according to our prior sensitivity analyses (see Supplementary Materials Fig. S1). Positive effect sizes for accuracy reflect cognitive enhancing effects of rTMS, while negative effect sizes for reaction times represent better cognitive effects after rTMS administration.

For studies where multiple outcome measures for a given task were reported, we selected the effect size of the primary outcome measure as defined by the authors (Begemann et al., 2020). If the primary outcome measure for a particular task was not defined, we included the most relevant measure according to our predefined cognitive domains. Where there existed several equally relevant outcome measures, we averaged the effect sizes across the multiple outcome measures (Chou et al., 2020; Martin et al., 2016, 2017; Sloan et al., 2020). For studies where multiple cognitive tasks assessed the same domain, outcomes across different tasks were averaged to generate domain-specific aggregate effect sizes. 66.7% (26/39) of studies had aggregated effect sizes in the accuracy dataset and for the reaction time dataset, 65.7% (23/35) of studies had aggregated effect sizes. All analyses were conducted using R version 4.0.2 (R Core Team, 2020), RStudio software version 1.3.1073 (RStudio Team, 2020) and ‘*meta*’, ‘*metafor*’ packages. Accuracy and reaction time outcomes were investigated in separate meta-analyses using random effects models with the Paule and Mandel (PM) estimator (Veroniki et al., 2016).

Heterogeneity was estimated using Cochran's Q (the ratio of the observed variation to the within-study error), τ^2^ (estimated amount of total heterogeneity), and I^2^ (total heterogeneity) statistics. I^2^ statistics was the primary measure of heterogeneity, with values of I^2^ on the order of 25%, 50% and 75% considered as indicating low, moderate, and high heterogeneity respectively (Higgins et al., 2003). Publication bias was assessed by funnel plots and the Egger’s test (Egger et al., 1997). Separate meta-analyses were conducted for accuracy and reaction times for all cognitive domains collapsed (i.e., analyses include all cognitive domains and produced overall effect sizes for both accuracy and reaction time) and for each of the six cognitive domains (attention, memory, motor, language, perception, and executive function) for excitatory and inhibitory offline HF-rTMS protocols (cTBS). Meta-analyses were only performed if the data consisted of at least three studies. Secondary subgroup analyses were performed for pulse frequency, the number of sessions, targeting method, and type of control condition. To decrease heterogeneity, sensitivity analyses were performed following the exclusion of outlier studies. These were defined as studies with a confidence interval that did not overlap with the confidence interval of the overall effect size (Harrer et al., 2019; Viechtbauer & Cheung, 2010).

## Results

### Search Results

53 studies and 54 experiment arms were included for quantitative analysis. Figure 1 summarises the search and screening process.

### Risk of bias Results

**Fig. 2:**
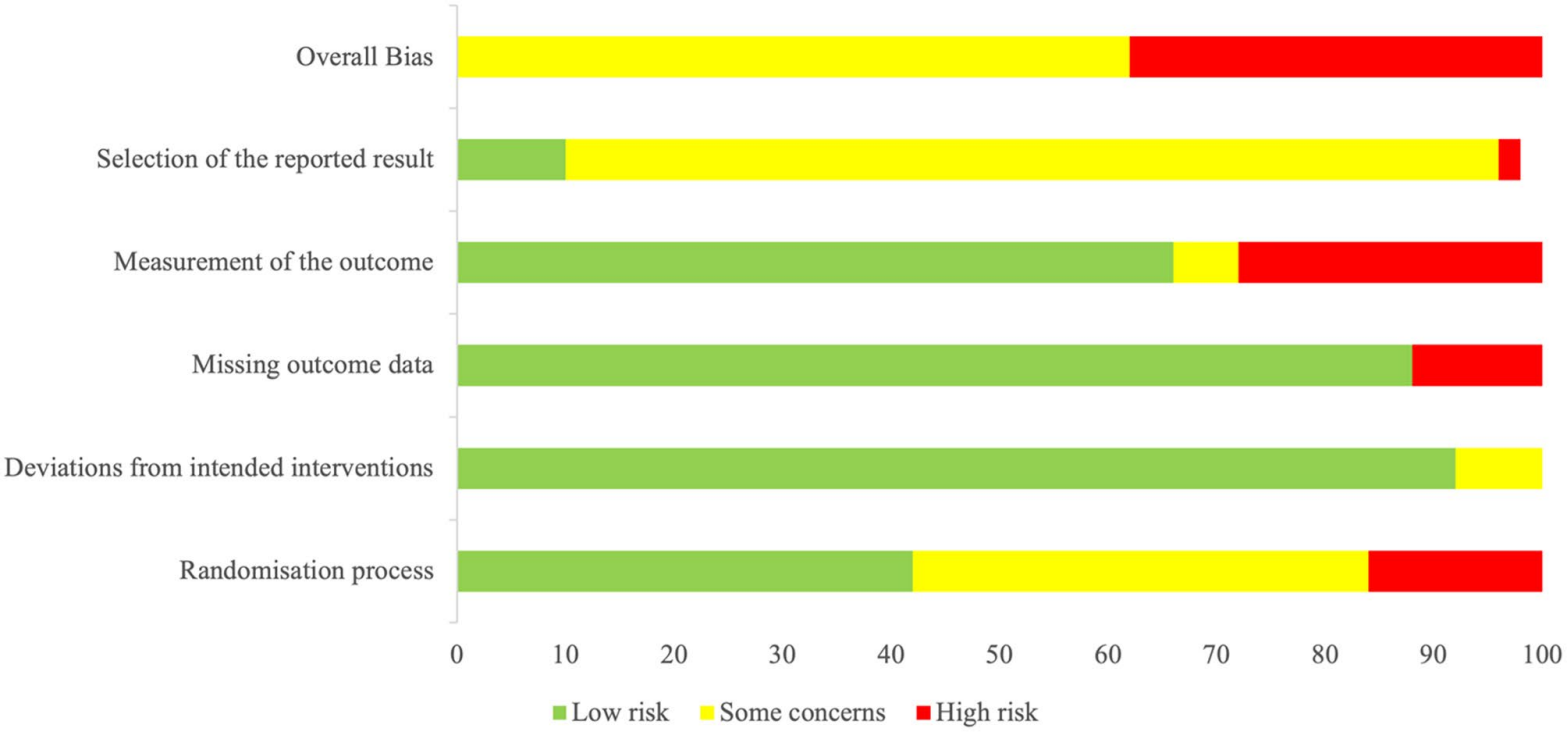
Summary plot of risk of bias assessment

For overall bias, 38.0% of studies had a high risk of bias and the rest (62.0%) had some concerns. Of note, the majority of studies had a low risk of bias due to deviations from intended interventions (92.0%) and missing outcome data (88.0%). All the studies used randomisation or counterbalance measures in group or trial allocation. However, the risk of bias tool requires detailed reporting of randomisation and allocation concealment. As 58.0% of studies did not provide sufficient information they were classified as presenting some concerns or a high risk in the randomisation process. 34.0% of studies did not report details about blinding measurements, which might affect the assessors’ ratings and led to some concerns or a high risk in measurement of outcomes. As most studies were not registered as clinical trials, 88.0% had some concerns or a high risk for selection of reported outcome (see Fig. 2).

### Study Characteristics

Table 2 shows the study characteristics for included studies. Twenty-six (49.1%) used a parallel study design, and twenty-eight studies (52.8%) used a crossover design. The total sample size of participants was N = 1507 and the mean age of participants across conditions ranged from 19.0 to 78.6 years. Across all domains, most offline HF-rTMS studies assessed effects on executive function (k = 29, 54.7%), followed by perception (k = 13, 24.5%) and attention (k = 21, 39.6%). Fewer studies investigated the effects of HF-rTMS for memory (k = 8, 12.1%), motor (k = 6, 15.1%), and language (k = 1, 1.9%) domains. Table 3 shows the rTMS parameters for included studies. Stimulation sites included cerebral (frontal: k = 36, 67.9%; parietal: k = 13, 24.5%; temporal: k = 3, 5.7%; occipital: k = 5, 9.4%) and cerebellar (k = 1, 1.9%) regions. The percentage of 5 Hz, 6 Hz, 10 Hz, 20 Hz, 25 Hz, iTBS, cTBS were respectively 7.5% (k = 4), 1.9% (k = 1), 32.1% (k = 17), 9.4% (k = 5), 1.9% (k = 1), 28.3% (k = 15), and 49.1% (k = 26). The figure-of-eight coil was used most frequently (k = 50, 94.3%), and only three studies (5.7%) used a circular coil. Half of studies used angular rotation (k = 29, 54.7%) and 30.2% of studies used an active control site (k = 16, 30.2%); additionally, 37.7% (k = 20) of studies used either a sham coil, spacer or stimulation intensity set to 0% of machine output.

**Table 2:** Characteristics of studies included in the meta-analysis

Study	Exp	Study design	Sample size		Age (M, SD)	Gender (M/F)	Cognitive tasks	Cognitive domains	Outcome measures
			A	C					
Allen et al. (2014)	1	Cross-over	16	16	27.4 (3.6)	11/5	Conscious detection task	Pe	AC, RT
Amiaz et al. (2011)		Parallel	10	10	A: 23.7 (3.1) C: 24.5 (3.6)	9/11	Filling-in task	Pe	AC
Baeken et al. (2012)		Cross-over	18	18	LDLPFC: 21.2 (1.4) RDLPFC: 24.5 (2.9)	0/18	Fitts’ paradigm	Mo	RT
Bagherzadeh et al. (2016)		Parallel	15	15	A: 39.1 (4.1)	10/20	CANTAB: DSP, S2B, PRM, SRM, SSP, SOC	EF, Me	AC, RT
					C: 34.5 (3.4)				
Banissy et al. (2010)	1	Cross-over	10	10	20–30	4/6	Emotion discrimination task	Pe	AC, RT
	2	Cross-over	10	10	20–35	5/5	Identity discrimination task	Pe	AC, RT
	3	Cross-over	6	6	21–36	4/2	Nonverbal auditory emotion recognition	Pe	AC
Barr et al. (2009)		Parallel	11	11	34.2 (7.2)	11/11	0-back, 1-back, 2-back, 3-back	At, EF	AC, RT
Chakraborty et al. (2021)		Cross-over	15	15	27 (3.0)	8/7	Multiple object tracking	At	AC
Chechlacz et al. (2015)		Cross-over	30	30	26.2 (4.9)	13/17	Free visual exploration task	At	RT
Cheng et al. (2013)		Cross-over	11	11	A: 24 (5.4) C: 22.6 (5.2)	7/13	Order and quantity tasks	Pe	RT
Choi et al. (2016)		Parallel	12	8	26.5 (4.6)	8/12	Sensory discrimination measurement	Mo	AC, RT
Chung et al. (2018)		Cross-over	18	18	25.6 (7.0)	8/10	2-back task, 3-back task	EF	AC
Clerget et al. (2011)		Parallel	12	12	25.1 (3.5)	18/6	Motor sequency experiment	Mo	AC, RT
De Raedt et al. (2010)		Cross-over	16	16	-	0/19	Exogenous cueing task (ECT)	At	RT
		Parallel	19	17	-	0/32	ECT	At	RT
Deng et al. (2022)		Cross-over	22	22	21 (2.4)	12/18	Change detection task	EF	AC
Dietrich et al. (2018)		Parallel	18	18	A: 30.4 (9.0) C: 29.2 (11.2)	36/0	Sentence repetition task	La	AC
Galea et al. (2010)		Parallel	LDLPFC: 10,	10	LDLPFC: 23.1 (3.7),	15/15	Serial reaction time task	Me	AC, RT
			RDLPFC: 10		RDLPFC: 23.3 (3.1) C: 24.5 (4.3)				
Gao et al. (2021)	2	Cross-over	16	16	21.13 (1.9)	9/7	Stroop, Associative memory test	At, EF, Me	AC, RT
Gaudeau-Bosma et al. (2013)		Parallel	9	10	31.6 (10.6)	11/8	0-back, 1-back, 2-back, 3-back	At, EF	AC
Huang et al. (2004)		Cross-over	24	24	27.0 (4.7)	12/12	Go/NoGo task	EF	RT
Ji et al. (2019)	1	Cross-over	5 Hz: 20	20		9/11		EF	RT
			25 Hz: 20 iTBS: 20				Stop signal task (SST)		
					21.6 (1.0)				
	2	Cross-over	18	18	22.1 (2.5)	13/5	SST	EF	RT
Kaderali et al. (2015)	2	Cross-over	8	8	-	-	Achromatic and chromatic motion discrimination task	Pe	AC
	3	Cross-over	8	8	-	-	Chromatic and achromatic detection task	Pe	AC
Kalla et al. (2009)		Cross-over	12	12	28.3	8/4	Visual search task	Pe	AC
Kazemi et al. (2020)		Cross-over	6 Hz: 10 8 Hz: 8	20	32.47 (11.0)	13/7	Rapid visual information processing task	At	AC, RT
Kim et al. (2012)		Parallel	8	8	63.13 (4.9)	-	Stroop task	At, EF	AC, RT
Kim et al. (2020)		Parallel	14	13	27.26 (2.4)	16/11	Auditory continuous performance test (CPT), visual CPT	At	RT
Leyman et al. (2009)	1	Cross-over	17	17	21.1 (1.5)	0/17	Negative Affective Priming (NAP) task	EF	RT
	2	Cross-over	22	22	24 (2.3)	0/22	NAP	EF	RT
Li et al. (2017)		Parallel	25	25	26.8 (1.4)	24/26	Stroop task	At, EF	AC, RT
Li et al. (2021)		Parallel	33	33	A: 25.8 (2.4) C: 25.6 (2.4)	36/30	0-back, 1-back, 2-back, Stroop	EF	AC, RT
Liang et al. (2021)		Parallel	LpSTS: 38, LDLPFC: 35	36	21.4 (2.5)	50/59	Self-matching task	At, EF	AC, RT
Meehan et al. (2013)	1	Parallel	11	11	A: 24.0 (2.9) C: 23.0 (2.3)	A: 6/5 C: 8/3	Continuous tracking task	Mo	AC, RT
Morgan et al. (2013)		Cross-over	17	17	25.0	-	Orientation task, Colour task, Dual task	EF	AC, RT
Palaus et al. (2020)		Parallel	14	13	29.4 (6.3)	13/14	Reaction time tasks, Raven’s progressive matrices, 3-back, Mental rotation task, Digit span tasks, Stop-switching task	At, EF, Pe	AC, RT
Pearce et al. (2014)		Parallel	10	10	27.4 (7.4)	10/10	Spatial working memory	EF	AC, RT
Pinto et al. (2018)		Parallel	iTBS:10, cTBS:10, iTBS:10, cTBS:10	10	22.8 (2.0)	20/31	Oddball paradigm	At	RT
Pinto et al. (2021)		Parallel	iTBS: 9, cTBS: 9	10	22.6 (2.3)	16/12	Oddball task, Trial making test, Stroop	At, EF	AC
Rahnev et al. (2013)	2	Cross-over	9	9	19–26	3/6	Perceptual discrimination task	Pe	AC
Schaller et al. (2013)		Parallel	21	17	A: 24.4 (2.7) C: 24.1 (2.9)	38/0	VFT, RFFT TAP: alertness, go/no-go, divided attention, working memory, flexibility	At, EF	AC, RT
Tambini et al. (2017)		Cross-over	22	22	20.7	5/17	Memory test	Me	AC
Tomlinson et al. (2014)	1	Cross-over	10	10	19–35	3/7	Aiming task Memory task	Me	AC
	2	Cross-over	13	13	18–29	4/9	Memory task	Me	RT
	2	Cross-over	9	9	22–43	4/5	SVFP	EF	RT
Vanbellingen et al. (2020)		Cross-over	31	31	33.4 (14.4)	23/23	TULIA, Postural imitation test	Mo	AC
Vanderhasselt et al. (2006)		Cross-over	28	28	23 (4.4)	0/28	Stroop task	At, EF	RT
Vanderhasselt et al. (2007)		Cross-over	20	20	24 (2.6)	0/20	Stroop task	At, EF	RT
Vanderhasselt et al. (2010)		Cross-over	20	20	27.7 (2.7)	0/20	Reaction task	At	RT
Varnava et al. (2013)		Cross-over	24	24	23.7 (3.7)	12/12	Line bisection task, Landmark task	Pe	RT
Vékony et al. (2018)		Parallel	cTBS: 17 iTBS: 18	16	cTBS: 24.2 (2.8) iTBS: 25.3 (2.7) C: 21.3 (2.3)	cTBS: 11/6 iTBS: 10/8 C: 4/12	1-back, 2-back, 3-back	EF	AC
Vidal-Pineiro et al. (2014)		Parallel	12	12	71.8 (6.8)	12/12	Episodic memory task	Me	AC
Wang and Voss (2015)		Parallel	8	8	20–32	7/9	Face-cued word recall testing	Me	AC
Wang et al. (2020)		Parallel	20 Hz: 29 iTBS: 20	29	A: 24 (2.6) C: 22.4 (2.2)	A: 29/29 C: 16/13	Game of dice task, Risky gains task	EF	AC
Wu et al. (2021)		Parallel	20 Hz: 20 iTBS: 20	20	20 Hz: 23.8 (3.4), iTBS: 23.9 (2.8), C: 23.5 (3.0)	34/26	0-back, 1-back, 2-back 3-back, Wisconsin card sorting test	At, EF	AC, RT
Yang et al. (2018a)		Cross-over	23	23	23.6 (3.0)	23/0	Adjusting amount task, Information sampling task (IST) SST, IST	EF	AC
Yang et al. (2018b)		Cross-over	20	20	21.8 (1.9)	20/0	Nine-hole peg test,	EF	AC, RT
Zhang & Fong (2019)		Parallel	6	6	A: 25.3 (2.0), C: 26.3 (2.3)	A: 3/3, C: 4/2	Minnesota dexterity, Purdue pegboard test, Two-ball rotation task	Mo	AC
Zito et al. (2021)		Cross-over	20	20	27.2 (3.3)	10/10	Agency task	EF	AC

*A* Active condition; *C* Control condition; *LDLPFC* Left dorsolateral prefrontal cortex; *RDLPFC* Right dorsolateral prefrontal cortex; *cTBS* continuous theta-burst stimulation; *iTBS* Intermittent theta-burst stimulation; *CANTAB* Cambridge Neuropsychological Test Automated Battery; *DSP* Digit Span Task; *S2B* Spatial 2-back task; *PRM* Pattern Recognition Memory Task; *SRM* Spatial Recognition Memory Task; *SSP* Spatial Span Task; *SOC* Stockings of Cambridge task; *ECT* Exogenous cueing task; *SST* Stop Signal Task; *CPT* Continuous performance test; *NAP* Negative Affective Priming; *VFT* Verbal fluency tasks; *RFFT* Ruff Figural Fluency Test; *SVFP* Standard variable foreperiod paradigm; *TAP* Test for attentional performance; *TULIA* a validated, comprehensive test for gesture production; *IST* Information Sampling Task; *At* Attention; *Pe* Perception; *Mo* Motor; *Me* Memory; *La* Language; *EF* Executive function; *AC* accuracy; *RT* Reaction time

**Table 3:** Study intervention characteristics

Study	Exp	Stimulation sites	Targeting method	Pulse frequency	Intensity	Coil type	Number of sessions	Total pulses per session	Control
Allen et al. (2014)	1	V1	MRI	cTBS	80% RMT	Round	1	600	Spacer
Amiaz et al. (2011)		Left DLPFC	Scalp measurements	10 Hz	90% RMT	Fig-of-8	1	1000	Spacer
Baeken et al. (2012)		Left and right DLPFC	MRI	10 Hz	110% MT	Fig-of-8	1	1560	90°
Bagherzadeh et al. (2016)		Left DLPFC	10–20	10 Hz	100% RMT	Fig-of-8	10	600	45°
Banissy et al. (2010)	1	rPoG, rPM	fMRI	cTBS	80% MT	Fig-of-8	1	300	Vertex
	2	rPoG, rPM	fMRI	cTBS	80% MT	Fig-of-8	1	300	Vertex
	3	rPoG, rPM	fMRI	cTBS	80% MT	Fig-of-8	1	300	Vertex
Barr et al. (2009)		Right and left DLPFC	fMRI	20 Hz	90% RMT	Fig-of-8	2	600	90°
		Left PTC	MRI	cTBS	90% AMT	Fig-of-8	1	600	1 Hz, 100% RMT
Chakraborty et al. (2021)		Left and right MT +	fMRI	cTBS	100% AMT	Fig-of-8	1	600	-
Chechlacz et al. (2015)		Left and right IPS	MRI	cTBS	80% RMT	Fig-of-8	1	801	Sham coil
Cheng et al. (2013)		Left and right IPS	MRI	cTBS	40% MSO	Fig-of-8	1	300	Vertex
Choi et al. (2016)		Motor area	Hot spot	10 Hz	90% RMT	Fig-of-8	1	900	45°
Chung et al. (2018)		Left DLPFC	10–20	SHAM + iTBS	75% RMT	Fig-of-8	1	600	90°
		Left DLPFC	10–20	iTBS + iTBS	75% RMT	Fig-of-8	2	1200	90°
Clerget et al. (2011)		Left BA 44	MRI	cTBS	80% RMT	Fig-of-8	1	600	Vertex
De Raedt et al. (2010)		Left DLPFC	MRI	10 Hz	110% RMT	Fig-of-8	1	1560	90°
		Right DLPFC	MRI	10 Hz	110% RMT	Fig-of-8	1	1560	90°
Deng et al. (2022)		Left parietal lobe	MRI	iTBS	80% RMT	Fig-of-8	5	600	90°
Dietrich et al. (2018)		Left pre-SMA	MRI	cTBS	120% RMT	Fig-of-8	1	600	Left MOG, 40% MSO
Galea et al. (2010)		Left and right DLPFC	MRI	cTBS	80% AMT	Fig-of-8	1	600	Occipital cortex, 70% MSO
Gao et al. (2021)		Left IPL	fMRI	20 Hz	100%RMT	Fig-of-8	5	1600	PreSMA
Gaudeau-Bosma et al. (2013)		Left DLPFC	MRI	10 Hz	110% RMT	Fig-of-8	10	2000	Sham coil
Huang et al. (2004)		Left DLPFC	Scalp measurements	5 Hz	100% RMT	Fig-of-8	1	1600	90°
Ji et al. (2019)	1	Right pre-SMA	MRI	5 Hz	110% RMT	Fig-of-8	1	1800	Sham coil
	1	Right pre-SMA	MRI	25 Hz	110% RMT	Fig-of-8	1	1800	Sham coil
	1	Right pre- SMA	MRI	iTBS	70% RMT	Fig-of-8	3	1800	Sham coil
	2	Right pre- SMA	MRI	iTBS	70% RMT	Fig-of-8	1	1800	Sham coil
Kaderali et al. (2015)	2	hMT +	MRI	cTBS	45% MSO	Fig-of-8	1	600	Vertex
	3	V1/V2	MRI	cTBS	45% MSO	Fig-of-8	1	600	Vertex
Kalla et al. (2009)		Right DLPFC and right V5	MRI	iTBS	40% MSO	Fig-of-8	1	600	Vertex
Kazemi et al. (2020)		Right DLPFC	10–20 EEG system	6 Hz	120% RMT	Fig-of-8	1	5400	90°
		Right DLPFC	10–20 EEG system	10 Hz	120% RMT	Fig-of-8	1	6000	90°
Kim et al. (2012)		Left DLPFC	10–20 EEG system	10 Hz	30% MSO	Fig-of-8	5	780	90°
Kim et al. (2020)		Left DLPFC	10–20 EEG system	10 Hz	80% RMT	Fig-of-8	1	1500	90°
Leyman et al. (2009)	1	Left DLPFC	MRI	10 Hz	110% RMT	Fig-of-8	1	1560	90°
Li et al. (2017)		Left DLPFC	Scalp measurements	10 Hz	110% RMT	Fig-of-8	7	1350	90°
		Left DLPFC	Scalp measurements	10 Hz	80% RMT	Fig-of-8	2	2000	90°
Li et al. (2021)		Left pSTS	10–20 EEG system	cTBS	80% AMT	Fig-of-8	1	600	90°
Liang et al. (2021)		Left DLPFC	10–20 EEG system	cTBS	80% AMT	Fig-of-8	1	600	90°
Meehan et al. (2013)		Left PMd	MRI	5 Hz	110% RMT	Fig-of-8	4	1200	Sham coil
Morgan et al. (2013)		Right PC, Left IFG	MRI	cTBS	80% AMT	Fig-of-8	1	600	45°
Palaus et al. (2020)		Right DLPFC	MRI	iTBS	80% AMT	Fig-of-8	10	600	90°
Pearce et al. (2014)		Right PPC	10–20 EEG system	5 Hz	80% RMT	Fig-of-8	6	300	Active control
Pinto et al. (2018)		Left and right DLPFC	Scalp measurements	iTBS cTBS	80% AMT	Fig-of-8	1	600	90°
Pinto et al. (2021)		Left DLPFC	Scalp measurements	iTBS cTBS	80% AMT	Fig-of-8	1	600	90°
Rahnev et al. (2013)	2	Occipital cortex	Hotspot	cTBS	80% PT	Fig-of-8	1	600	Sham: 90° Control: Pz
Schaller et al. (2013)		Left DLPFC	Scalp measurements	25 Hz	100% RMT	Round	9	750	Sham coil
Tambini et al. (2017)		pIPC	fMRI	cTBS	80% AMT	Fig-of-8	1	600	S1
Tomlinson et al. (2014)	1	Left mid cerebellar hemisphere	Scalp measurements	cTBS	80% AMT	Fig-of-8	1	600	Right mid cerebellar hemisphere
	2	Left lateral cerebellar hemisphere	Scalp measurements	cTBS	80% AMT	Fig-of-8	1	600	Right lateral cerebellar hemisphere
Vanbellingen et al. (2020)		Left IFG and right IPL	10–20 EEG system	cTBS	80% RMT	Round	1	801	Sham coil
Vanderhasselt et al. (2006)		Left DLPFC	MRI	10 Hz	110% RMT	Fig-of-8	1	1560	90°
Vanderhasselt et al. (2007)		Right DLPFC	MRI	10 Hz	110% RMT	Fig-of-8	1	1560	90°
Vanderhasselt et al. (2010)		Left DLPFC	MRI	10 Hz	110% RMT	Fig-of-8	1	1560	90°
Varnava et al. (2013)		Left and right AG	MRI	cTBS	80% RMT	Fig-of-8	1	600	90°
Vékony et al. (2018)		Left and Right DLPFC	MRI	cTBS iTBS	30% MSO	Fig-of-8	2	600	45°
Vidal-Pineiro et al. (2014)		Left IFG	MRI	iTBS	80% AMT	Fig-of-8	1	600	Sham coil
Wang and Voss (2015)		Lateral PC	MRI	20 Hz	100% RMT	Fig-of-8	5	1600	0% RMT
Wang et al. (2020)		Left DLPFC	MRI	20 Hz	90% RMT	Fig-of-8	2	1800	Sham coil
		Left DLPFC	MRI	iTBS	80% RMT	Fig-of-8	3	1800	Sham coil
Wu et al. (2021)		Left DLPFC	MRI	20 Hz	110% RMT	Fig-of-8	1	1800	Sham coil
		Left DLPFC	MRI	iTBS	70% RMT	Fig-of-8	3	600	Sham coil
Yang et al. (2018a)		Left DLPFC	Scalp measurements	iTBS	80% RMT	Fig-of-8	1	1800	Sham coil
Yang et al. (2018b)		Right IFG	Scalp measurements	10 Hz	100% RMT	Fig-of-8	1	900	Sham coil
Zhang & Fong (2019)		Right M1	Standard head in Neuronavigation	iTBS	80% RMT	Fig-of-8	4	600	Sham coil
Zito et al. (2021)		Right PPC	10–20 EEG system	cTBS	80% RMT	Fig-of-8	1	600	Vertex

*V1* Primary visual cortex; *DLPFC* Dorsolateral prefrontal cortex; *rPoG* Right postcentral gyrus; *rPM* Right lateral premotor cortex; *PPC* Posterior parietal cortex; *PTC* Posterior temporal cortex; *MT* + middle temporal area and medial superior temporal area; *IPS* Intraparietal sulcus; *BA 44* Brodmann area 44 (Broca’s area); *SMA* Supplementary motor area; *IPL* Inferior parietal lobule; *hMT* + The point that elicited the strongest moving phosphene; *V2* Secondary visual cortex; *V5* Middle temporal visual area; *pSTS* Posterior superior temporal sulcus; *PMd* dorsal premotor cortex; *PC* Parietal cortex; *pIPC* Posterior inferior parietal cortex; *AG* Angular gyrus; *IFG* Inferior frontal gyrus; *IFS* Inferior frontal sulcus; *MRI* Magnetic resonance imaging (including standard MRI scans and individualised MRI scans); *fMRI* Functional magnetic resonance imaging; *cTBS* continuous theta-burst stimulation; *iTBS* Intermittent theta-burst stimulation; *MSO* maximum stimulator output; *MT* Motor thresholds; *AMT* Active motor thresholds; *MSO* Maximum stimulator output; *PT* Phosphene threshold; *Fig-of-8* Figure-of-eight coil; *MOG* Middle occipital gyrus; *Pz* Parietal midline; *S1* Primary somatosensory cortex; *POz* Medial parietal cortex

### Accuracy

#### Cognitive Effects of Offline Excitatory HF-rTMS

##### All Cognitive Domains Collapsed

**Fig. 3:**
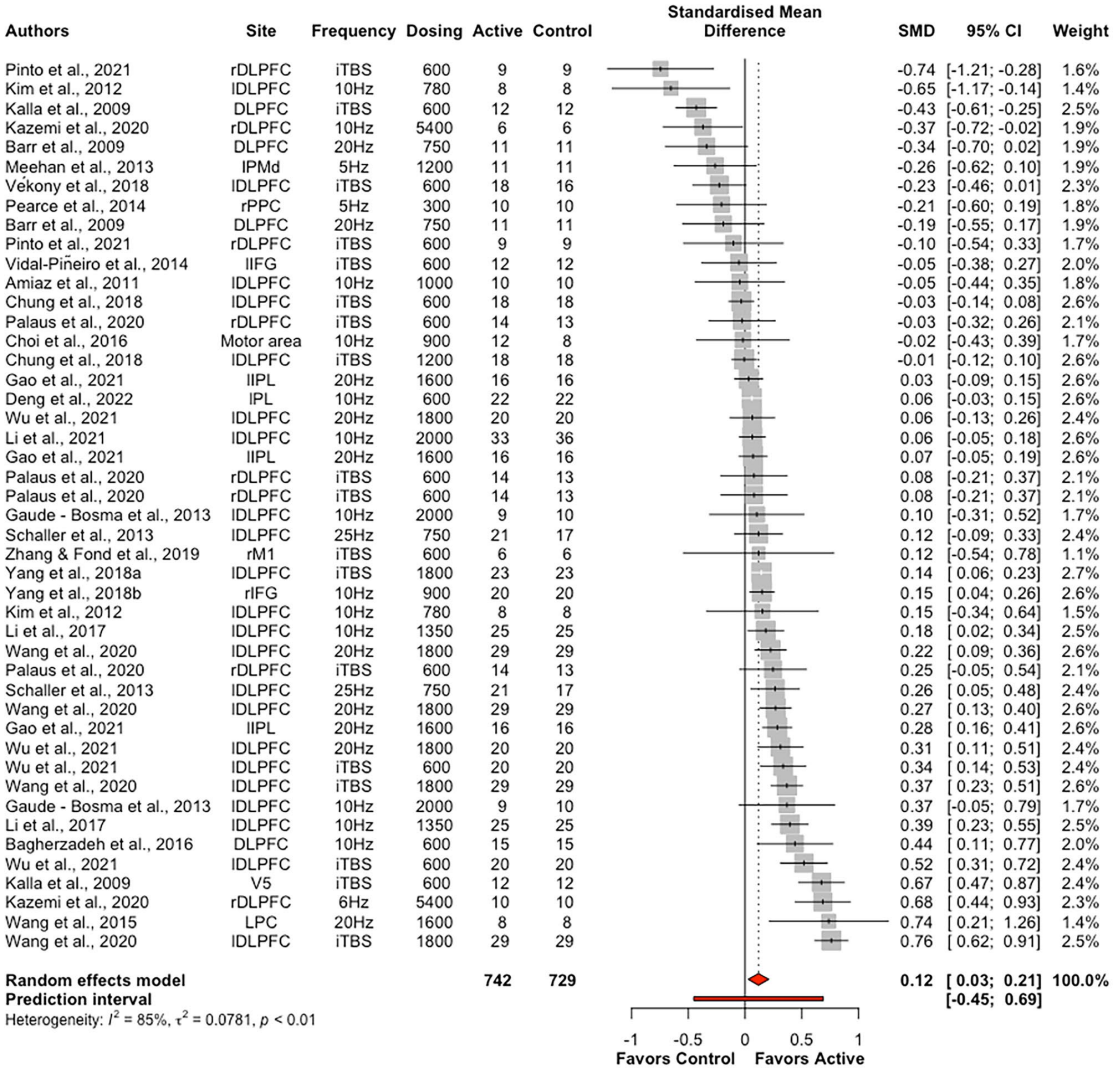
Forest plots of effects of excitatory HF-rTMS for accuracy. *Note*: Dosing represents the total pulses per session; l: Left; r: Right; DLPFC: Dorsolateral prefrontal cortex; PMd: dorsal premotor cortex; PPC: Posterior parietal cortex; IFG: Inferior Frontal Gyrus; IPL: Inferior parietal lobule; PL: Parietal lobe; M1: Primary motor cortex; PC: Parietal cortex; V5: Visual cortex

**Fig. 4:**
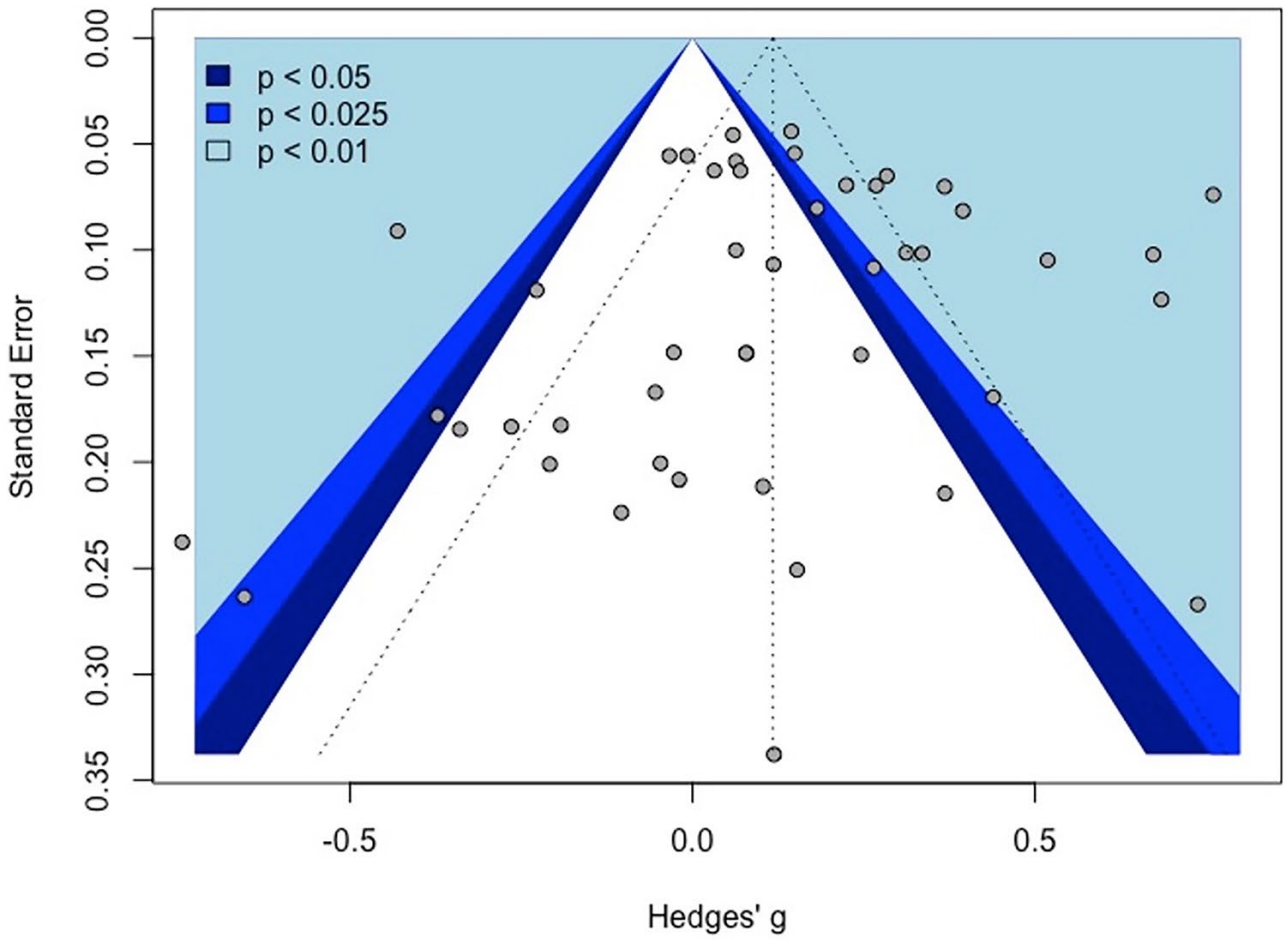
Funnel plot of excitatory HF-rTMS for accuracy

The overall effect of excitatory offline HF-rTMS (5 Hz, 6 Hz, 10 Hz, 20 Hz, 25 Hz, iTBS) on accuracy across all cognitive domains collapsed was statistically significant (k = 46, g = 0.12, 95% CI = [0.03; 0.21], *p* < 0.05) (see Table 4 and Fig. 3). The heterogeneity was substantial (*I*^2^ = 85.4%, ${\tau }^{2}$= 0.08, Q = 307.69, *p* < 0.001). Following removal of thirteen outlier studies, active HF-rTMS remained a significant small sized effect compared to control (k = 33, g = 0.12, 95% CI = [0.07; 0.16], *p* < 0.001); however, heterogeneity remained moderate *I*^2^ (48.7%). Visual inspection of the contoured funnel plot (see Fig. 4) and the Egger’s test (*p* = 0.54) revealed no publication bias.

**Table 4:** Summary table of meta-analysis results across cognitive domains for accuracy

Comparison	k	SMD	95% CI	τ^2^	Q	*I*^2^	*p*
Excitatory HF-rTMS							
Overall	46	0.12	[0.03; 0.21]	0.08	307.69	85.4%	0.01*
Attention	12	0.06	[-0.12; 0.25]	0.09	51.24	78.5%	0.52
Executive function	24	0.14	[0.03; 0.26]	0.07	171.26	86.6%	0.01*
Memory	3	0.29	[-0.13; 0.70]	0.11	6.86	70.8%	0.18
Motor	3	-0.12	[-0.37; 0.13]	0.00	1.35	0.00%	0.35
Perception	4	0.11	[-0.35; 0.58]	0.21	66.70	95.5%	0.64
Inhibitory HF-rTMS							
Overall	29	-0.05	[-0.18; 0.08]	0.12	555.63	95.0%	0.48
Attention	4	-0.25	[-0.53; 0.02]	0.07	40.79	92.6%	0.07
Executive function	6	-0.22	[-0.55; 0.12]	0.16	145.23	96.6%	0.21
Memory	3	0.16	[-0.41; 0.73]	0.23	56.33	96.4%	0.59
Motor	3	0.37	[-0.03; 0.77]	0.11	31.13	93.6%	0.07
Perception	12	-0.00	[-0.14; 0.12]	0.04	61.42	82.1%	0.97

*SMD* standardised mean difference; *CI* confidence interval; *τ*^2^ estimated amount of total heterogeneity; *Q* the ratio of the observed variation to the within-study error; *I*^2^ total heterogeneity; *p < 0.5

##### Attention

There was no significant effect of excitatory HF-rTMS (6 Hz: k = 1, 10 Hz: k = 4, 10 Hz: k = 3, 25 Hz: k = 1, iTBS: k = 3) on attention compared to control (k = 12, g = 0.06, 95% CI = [-0.12; 0.25], *p* = 0.52, see Table 4, Supplementary Materials Fig. S2).

##### Executive Function

**Fig. 5:**
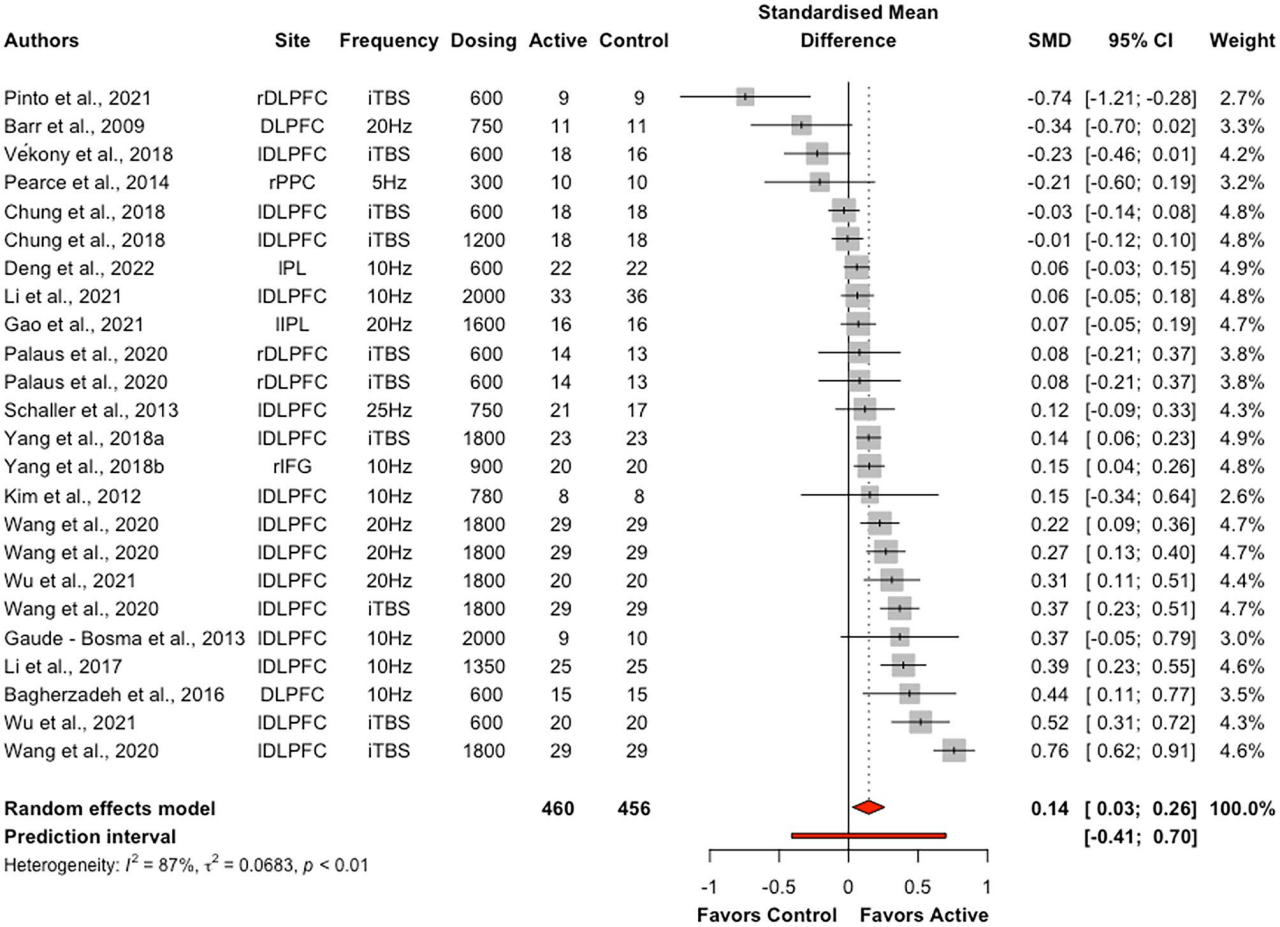
Forest plots for effects of excitatory HF-rTMS on executive function for accuracy. *Note*: Dosing represents the total pulses per session; l: Left; r: Right; DLPFC: Dorsolateral prefrontal cortex; PPC: Posterior parietal cortex; PL: Parietal lobe; IPL: Inferior parietal lobule; IFG: Inferior frontal gyrus

Active HF-rTMS (5 Hz: k = 1, 10 Hz: k = 7, 20 Hz: k = 5, 25 Hz: k = 1, iTBS: k = 10) had a significant positive effect on executive function (k = 24, g = 0.14, 95% CI = [0.03; 0.26], *p* < 0.05) with heterogeneity at 86.6% (see Table 4 and Fig. 5). After removing outliers, heterogeneity stayed at 48.7%.

##### Memory

No significant effect was found for the memory domain for active HF-rTMS (20 Hz: k = 2, iTBS: k = 1) relative to control groups (k = 3, g = 0.29, 95% CI = [-0.13; 0.70], *p* = 0.18, see Table 4, Supplementary Materials Fig. S2).

##### Motor

No significant effect was found for the motor domain for active HF-rTMS (5 Hz: k = 1, 10 Hz: k = 1, iTBS: k = 1) relative to control groups (k = 3, g = -0.12, 95% CI = [-0.37; 0.13], *p* = 0.35, see Table 4, Supplementary Materials Fig. S2).

##### Perception

No significant effect was found for the perception domain for active HF-rTMS (10 Hz: k = 1, iTBS: k = 3) relative to control groups (k = 4, g = 0.11, 95% CI = [-0.35; 0.58], *p* = 0.64, see Table 4, Supplementary Materials Fig. S2).

##### Language

As no excitatory HF-rTMS study examined effects on language, we did not perform meta-analyses in this domain.

#### Cognitive effects of offline inhibitory HF-rTMS

##### All Cognitive Domains Collapsed

As shown in Table 4, there was no significant effect of inhibitory offline HF-rTMS (cTBS) paradigms on accuracy (k = 29, g = -0.05, 95% CI = [-0.18; 0.08], *p* = 0.48, see Supplementary Materials Fig. S3).

##### Attention

No significant effect of cTBS was found for attention (k = 4, g = -0.25, 95% CI = [-0.53; 0.02], *p* = 0.07, see Table 4, Supplementary Materials Fig. S4).

##### Executive Function

No significant effect of cTBS was found for executive functioning (k = 6, g = -0.22, 95% CI = [-0.55; 0.12], *p* = 0.21, see Table 4, Supplementary Materials Fig. S4).

##### Memory

There was no significant effect of cTBS for memory (k = 3, g = 0.16, 95% CI = [-0.41; 0.73], *p* = 0.59, see Table 4, Supplementary Materials Fig. S4).

##### Motor

There was no significant effect of cTBS for motor (k = 3, g = 0.37, 95% CI = [-0.03; 0.77], *p* = 0.07, see Table 4, Supplementary Materials Fig. S4).

##### Perception

There was no significant effect of cTBS for perception (k = 12, g = -0.00, 95% CI = [-0.14; 0.12], *p* = 0.97, see Table 4, Supplementary Materials Fig. S4).

##### Language

Less than three studies investigated the effect of cTBS on language, thus meta-analyses were not conducted (see Supplementary Materials Fig. S4).

#### Subgroup Analyses

**Fig. 6:**
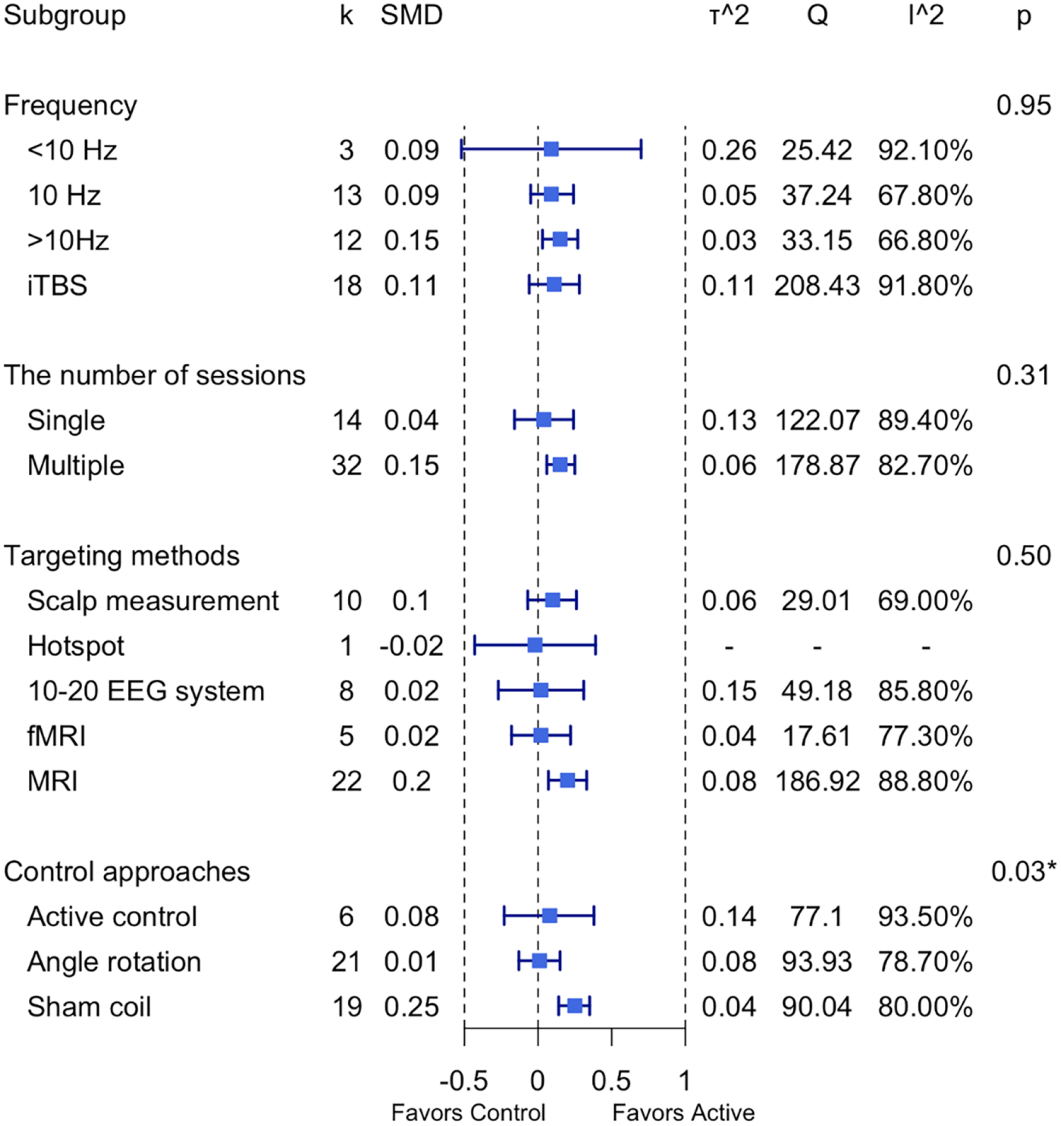
Summary plot of subgroup-analysis results for accuracy. *Note:* SMD: standardised mean difference; CI: confidence interval; τ^2^: estimated amount of total heterogeneity; Q: the ratio of the observed variation to the within-study error; I^2^: total heterogeneity; *p < 0.5

For all the cognitive domains collapsed, secondary subgroup analyses of excitatory protocols (HF-rTMS and iTBS) revealed a significant effect of control approaches (Active control: g = 0.08, Angle rotation: g = 0.01, Sham coil: g = 0.25, *p* < 0.05) (see Fig. 6 and Supplementary Materials Fig. S5). No significant differences were found for any other subgroup analyses (see Fig. 6).

### Reaction Time

#### Cognitive Effects of Offline Excitatory HF-rTMS

##### All Cognitive Domains Collapsed

**Fig. 7:**
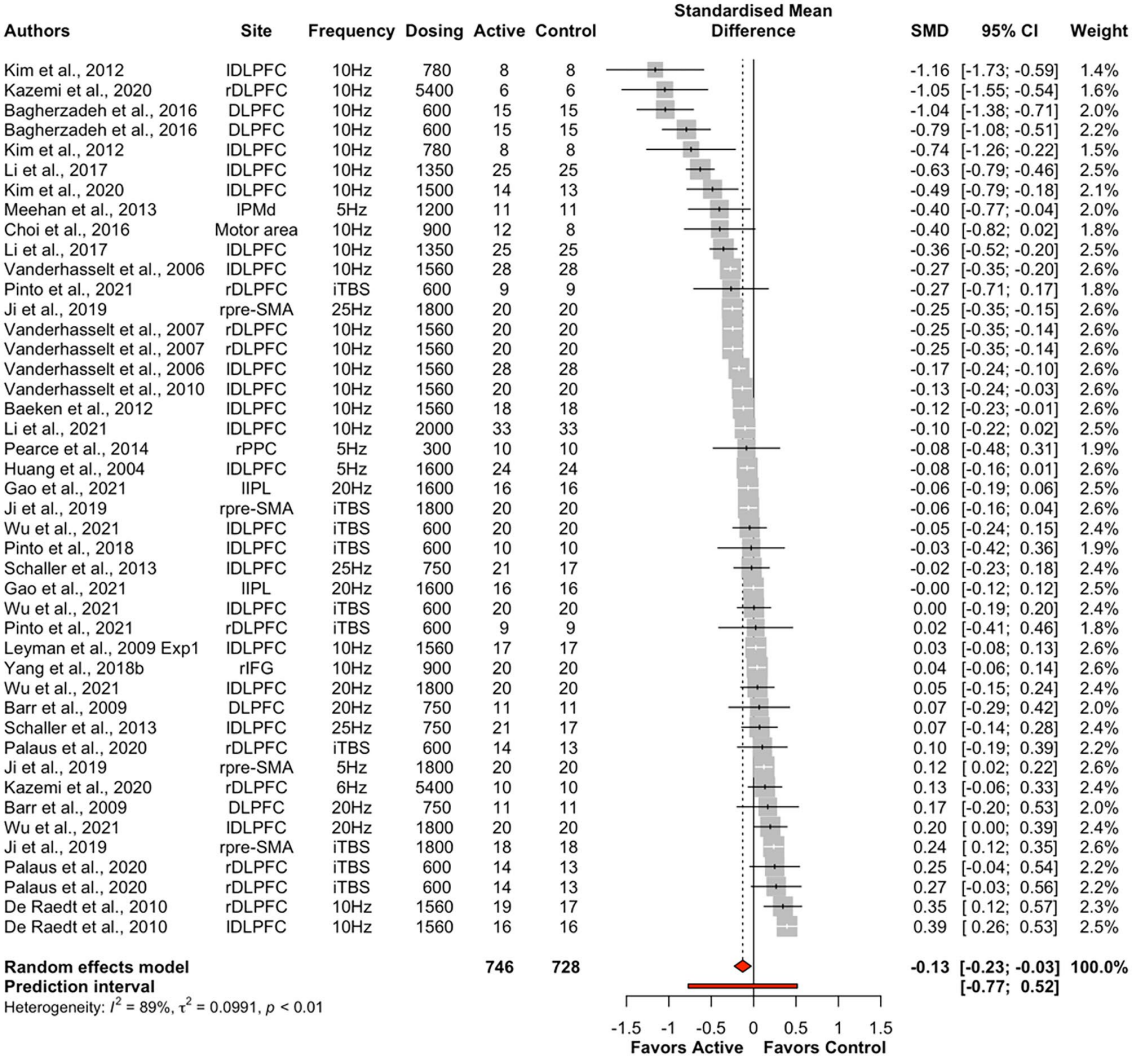
Forest plots of effects of excitatory HF-rTMS for reaction time. *Note*: Dosing represents the total pulses per session; l: Left; r: Right; b: Bilateral; DLPFC: Dorsolateral prefrontal cortex; PMd: dorsal premotor cortex; SMA: Supplementary motor area; PPC: Posterior parietal cortex; IPL: Inferior parietal lobule; IFG: Inferior frontal gyrus

**Fig. 8:**
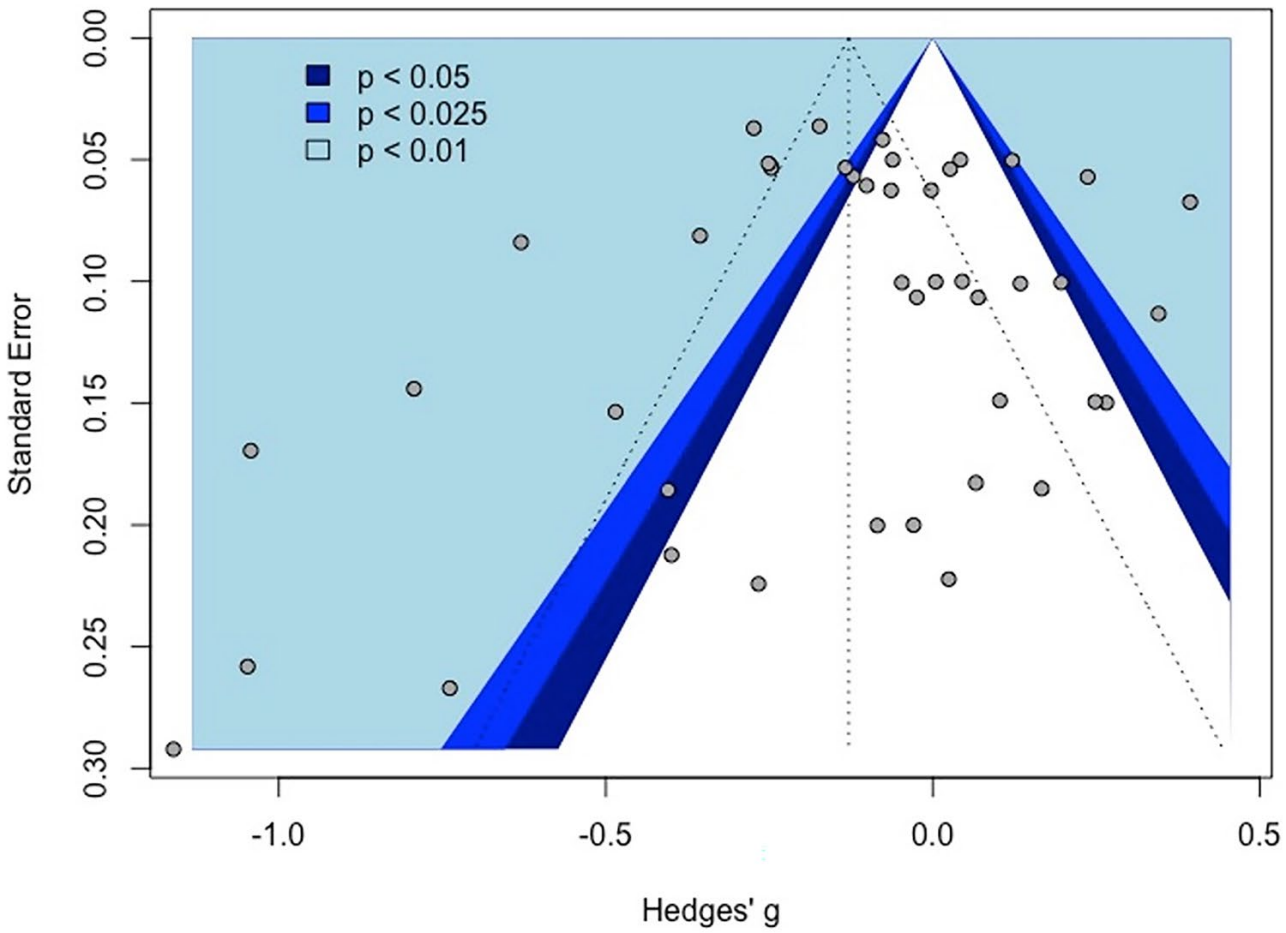
Funnel plot of excitatory HF-rTMS for reaction time

Excitatory offline HF-rTMS (5 Hz, 6 Hz, 10 Hz, 20 Hz, 25 Hz, iTBS) was associated with significantly reduced reaction times relative to control (k = 44, g = -0.13, 95% CI = [-0.23; -0.03], *p* < 0.05; see Table 5 and Fig. 7)*.* However, heterogeneity was large (*I*^*2*^ = 88.5%, *p* < 0.001). Following removal of eleven outlier studies, active HF-rTMS still showed a significant small sized effect compared to control (k = 33, g = -0.10, 95% CI = [-0.16; -0.04], *p* < 0.001) with heterogeneity moderate *I*^2^ (71.8%). Visual inspection of the contoured funnel plot (see Fig. 8) revealed some evidence of potential risk of publication bias, however, this was not corroborated by the Egger’s test (*p* = 0.63).

**Table 5:** Summary table of meta-analysis results across cognitive domains for reaction time

Comparison	k	SMD	95% CI	τ^2^	Q	*I*^2^	*p*
Excitatory HF-rTMS							
Overall	44	-0.13	[-0.23; -0.03]	0.10	375.39	88.5%	0.01*
Attention	18	-0.10	[-0.29; 0.09]	0.15	182.06	90.7%	0.30
Executive function	21	-0.11	[-0.21; -0.01]	0.04	151.44	86.8%	0.03*
Motor	3	-0.22	[-0.41; -0.04]	0.01	3.47	42.4%	0.02*
Inhibitory HF-rTMS							
Overall	19	-0.01	[-0.09; 0.12]	0.04	81.24	77.8%	0.80
Attention	5	-0.07	[-0.16; 0.02]	0.00	6.41	37.6%	0.12
Executive function	6	-0.02	[-0.17; 0.12]	0.03	27.82	82.0%	0.74
Perception	6	0.07	[-0.09; 0.23]	0.03	20.25	75.3%	0.40

*SMD* standardised mean difference; *CI* confidence interval; *τ*^2^ estimated amount of total heterogeneity; *Q* the ratio of the observed variation to the within-study error; *I*^2^: total heterogeneity; *p < 0.5

##### Attention

There was no significant effect of excitatory HF-rTMS (6 Hz: k = 1, 10 Hz: k = 9, 20 Hz: k = 3, 25 Hz: k = 1, iTBS: k = 4) on attention compared to control (k = 18, g = -0.10, 95% CI = [-0.29; 0.09], *p* = 0.30, see Table 5, Supplementary Materials Fig. S6).

##### Executive Function

**Fig. 9:**
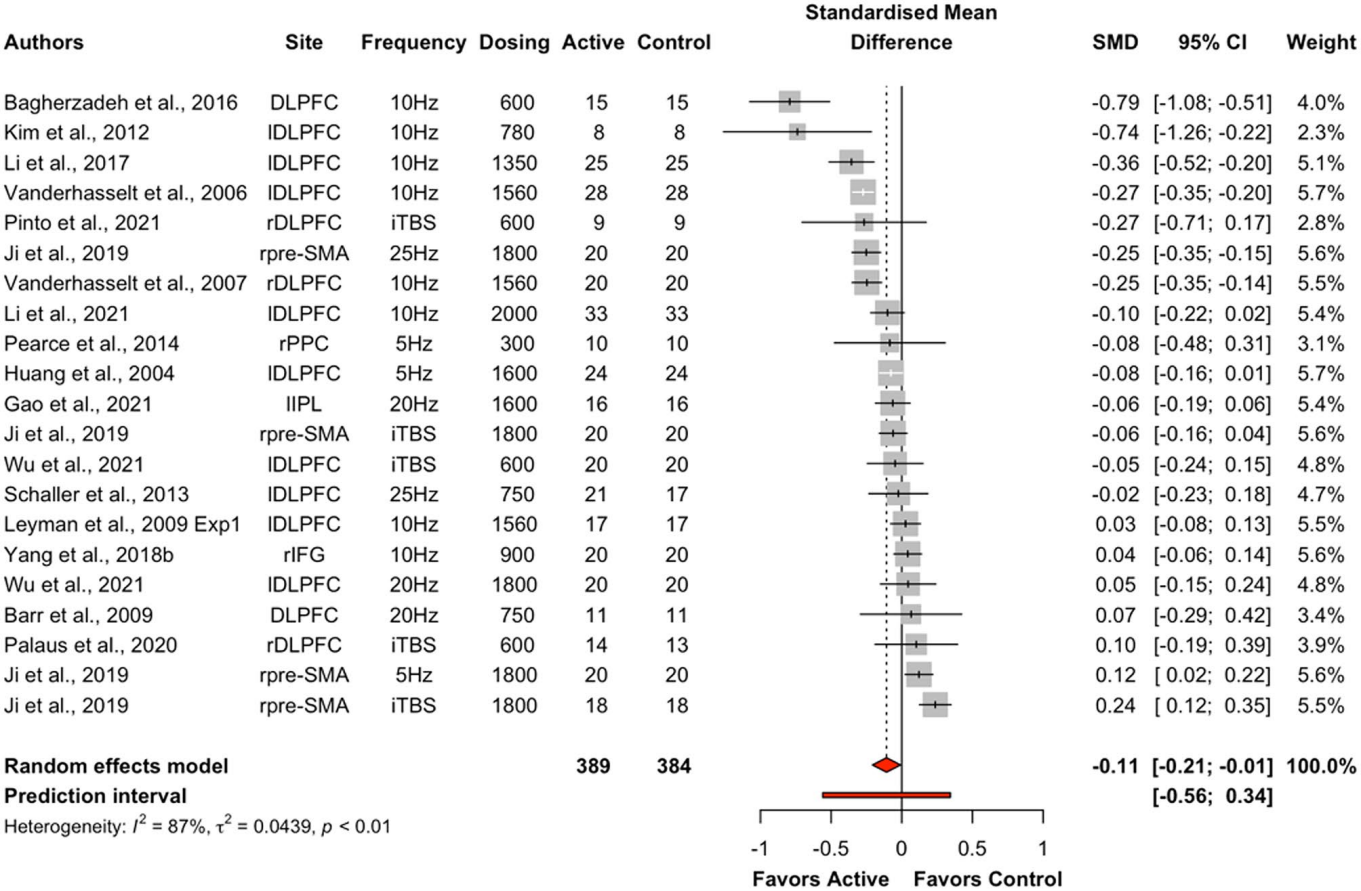
Forest plots for effects of excitatory HF-rTMS on executive function for reaction time. *Note*: Dosing represents the total pulses per session; l: Left; r: Right; b: Bilateral; DLPFC: Dorsolateral prefrontal cortex; SMA: Supplementary motor area; PPC: Posterior parietal cortex; IPL: Inferior parietal lobule; IFG: Inferior frontal gyrus

Active HF-rTMS (5 Hz: k = 3, 10 Hz: k = 8, 20 Hz: k = 3; 25 Hz: k = 2; iTBS: k = 5) had a significant effect on executive function (k = 21, g = -0.11, 95% CI = [-0.21; -0.01], *p* < 0.05) with heterogeneity at 86.8% (see Table 5 and Fig. 9).

##### Motor

**Fig. 10:**
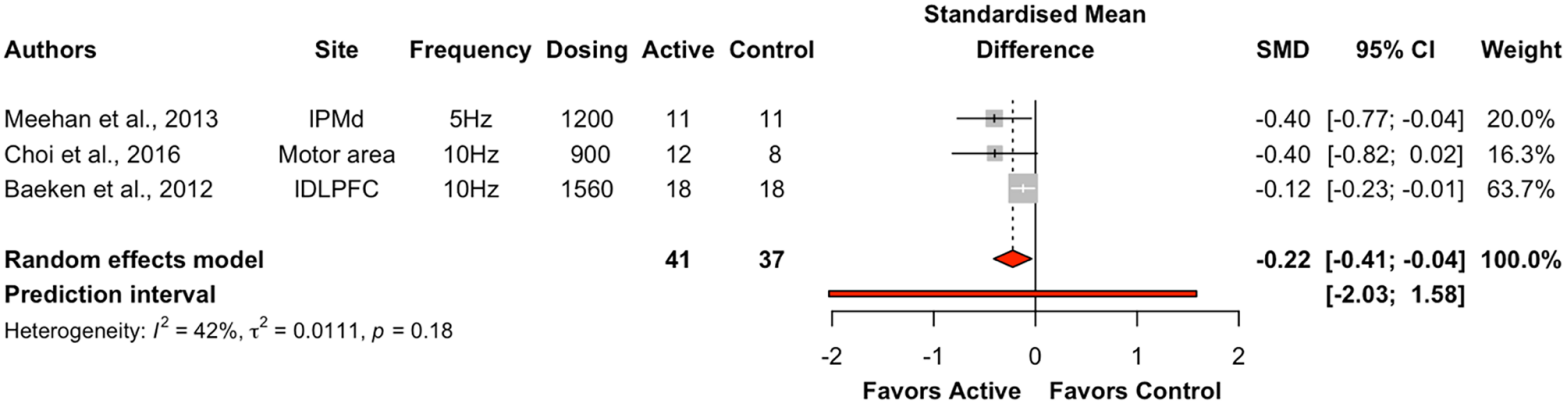
Forest plots for effects of excitatory HF-rTMS on motor for reaction time. *Note*: Dosing represents the total pulses per session; l: Left; r: Right; b: Bilateral; PMd: dorsal premotor cortex; DLPFC: Dorsolateral prefrontal cortex

Active HF-rTMS (5 Hz: k = 1, 10 Hz: k = 2) had a significant effect on the motor domain (k = 3, g = -0.22, 95% CI = [-0.41; -0.04], *p* < 0.05) with heterogeneity at 42.4% (see Table 5, Fig. 10).

##### Language, Memory, and Perception

As there were fewer than three studies investigating the effect of rTMS on language, memory, and perception (see Table 5, Supplementary Materials Fig. S6), meta-analyses were not conducted.

#### Cognitive Effects of Offline Inhibitory HF-rTMS

##### All Cognitive Domains Collapsed

For cTBS paradigms (k = 19), no significant effect was found for active HF-rTMS compared to control (k = 19, g = -0.01, 95% CI = [-0.09; 0.12], *p* = 0.80, see Table 5, Supplementary Materials Fig. S7).

##### Attention

There was no significant effect of cTBS on attention (k = 5, g = -0.07, 95% CI = [-0.16; 0.02], *p* = 0.12, see Table 5, Supplementary Materials Fig. S8).

##### Executive Function

Inhibitory cTBS paradigms yielded no significant effects on executive function (k = 6, g = -0.02, 95% CI = [-0.17; 0.12], *p* = 0.74, see Table 5, Supplementary Materials Fig. S8).

##### Perception

There was no significant effect of cTBS on perception (k = 6, g = 0.07, 95% CI = [-0.09; 0.23], *p* = 0.40, see Table 5, Supplementary Materials Fig. S8).

##### Language, Memory, and Motor

As less than three studies probed the effects of cTBS on the language, memory and motor domains (see Table 5, Supplementary Materials Fig. S8), meta-analyses were not performed.

#### Subgroup Analyses

**Fig. 11:**
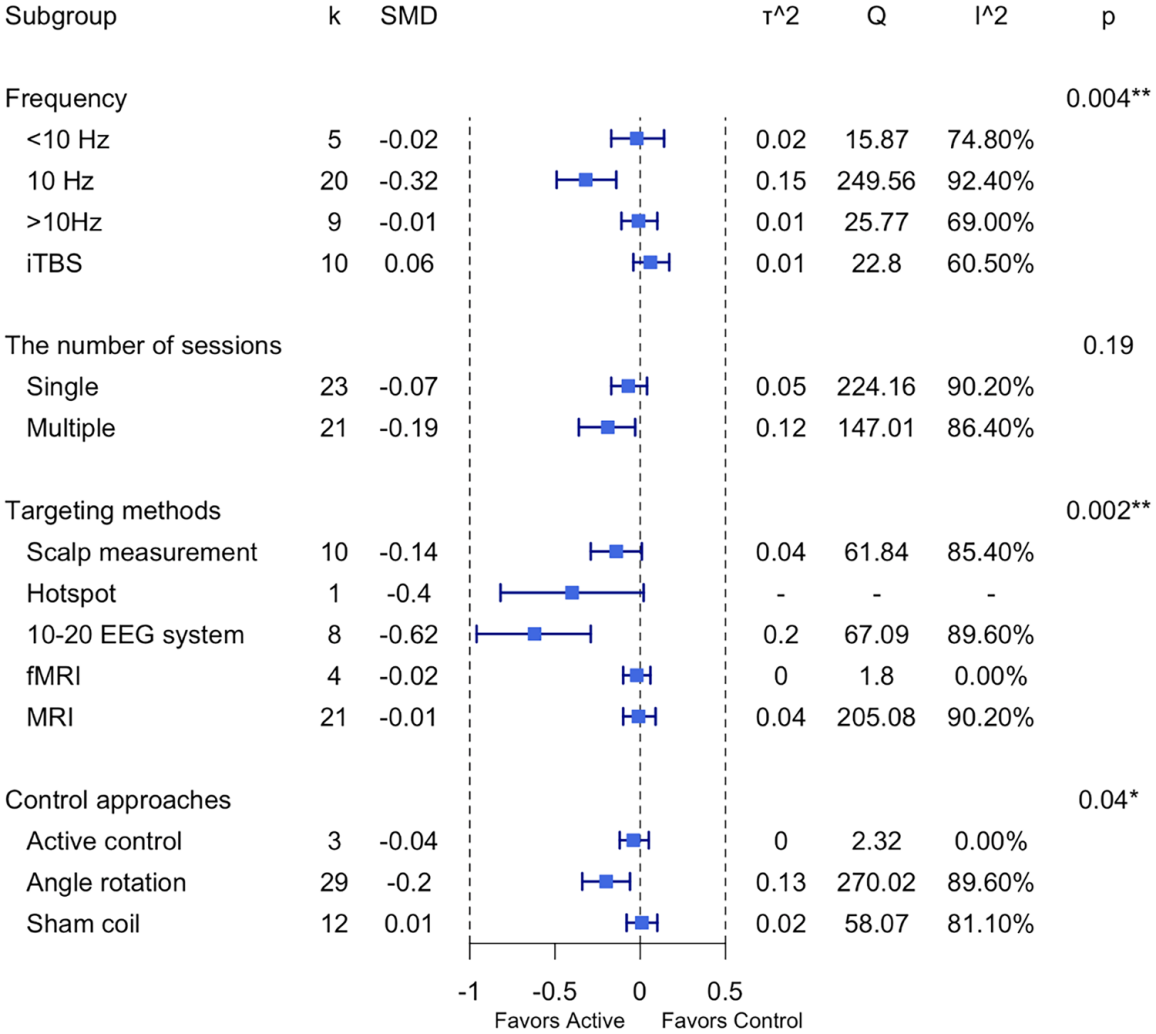
Summary plot of subgroup-analysis results for reaction time. *Note:* SMD: standardised mean difference; CI: confidence interval; τ^2^: estimated amount of total heterogeneity; Q: the ratio of the observed variation to the within-study error; I^2^: total heterogeneity; *p < 0.5, **p < 0.01

For all the cognitive domains collapsed, secondary subgroup analyses of excitatory protocols (HF-rTMS and iTBS) revealed that 10 Hz rTMS was relatively greater compared to other frequencies (< 10 Hz: g = -0.02, 10 Hz: g = -0.32, > 10 Hz: g = -0.01, iTBS: g = 0.06, *p* < 0.01) for improving reaction times (see Fig. 11, Supplementary Materials Fig. S9). There was also a significant effect of targeting methods (Scalp: g = -0.14, Hotspot: g = -0.40, 10–20 EEG system: g = -0.62, fMRI: g = -0.02, MRI: g = -0.01, *p* < 0.01) (see Fig. 11, Supplementary Materials Fig. S10). Control methods using angle rotation (i.e., 45°, 90°) demonstrated a relatively larger effect relative to other methods (Active control: g = -0.04, Angle rotation: g = -0.20, Sham coil: g = 0.01, *p* < 0.05; see Fig. 10 and Supplementary Materials Fig. S11). No significant differences were found for the number of sessions (see Fig. 11).

## Discussion

In this systematic review and meta-analysis, we aimed to clarify the cognitive effects of offline HF-rTMS on accuracy and reaction time performance in healthy participants. Across collapsed cognitive domains, excitatory offline HF-rTMS demonstrated overall small sized cognitive enhancing effects for both accuracy and reaction time. Although the direct of effects favoured active stimulation for most cognitive domains, the memory and motor domains contributed relatively larger effect sizes for accuracy and response times, respectively. A greater enhancing effect for HF-rTMS was found for executive function in accuracy and faster reaction times were demonstrated for both the executive functioning and motor domains. Overall effects were moderated by stimulation pulse frequency, control approaches and targeting methods.

The findings of this study extend evidence from several prior meta-analyses conducted in healthy and clinical cohorts (Begemann et al., 2020; Beynel et al., 2019; de Boer et al., 2021; Martin et al., 2016; Patel et al., 2020). A recent online rTMS meta-analysis found that HF-rTMS (i.e., 10 Hz and 20 Hz rTMS) showed reduced accuracy and slower reaction times across cognitive domains including executive function and motor domains in healthy populations (Beynel et al., 2019). Evidence from offline rTMS meta-analyses examining the effects of rTMS administered to the DLPFC across four cognitive domains, showed that excitatory stimulation resulted in a significant small sized improvement in executive functioning (SMD = 0.25), but not for working and episodic memory (Patel et al., 2020), which is in line with our findings. However, Patel’s and our current meta-analyses are constrained by the restricted evidence of offline HF-rTMS cognitive effects in targeted regions beyond the prefrontal cortex, which in turn may limit the interpretation of findings for some cognitive domains. Several studies in clinical samples (e.g., Alzheimer’s disease) and healthy groups, for example, have successfully targeted regions outside of the prefrontal cortex (e.g., precuneus, cortical-hippocampal networks) with offline HF-rTMS and observed cognitive improvement consistent with target-function alignment (Koch et al., 2018, 2022; Wang et al, 2014). In contrast to Patel and colleagues (2020), our study was not limited to DLPFC, included a larger number of studies, analysed both standard HF-rTMS and TBS, expanded to six cognitive domains, performed separate analyses on accuracy and reaction time, and explored moderators of cognitive effects of HF-rTMS.

Similar evidence for efficacy has also been provided from studies in neuropsychiatric samples. Specifically, a meta-analysis of the cognitive effects of rTMS in psychiatric disorders revealed a significant and moderate effect on working memory in patients with schizophrenia in a secondary analysis (SMD = 0.51) (Martin et al., 2016). A follow up meta-analysis conducted only on patients with depression revealed small sized effects on specific tasks involving executive functioning and processing speed (Martin et al., 2017). Likewise, across mixed diagnoses, offline rTMS treatment benefited neuropsychiatric patients (including schizophrenia, depression, dementia, Parkinson’s disease, multiple sclerosis, stroke, and traumatic brain injury) with a small effect on working memory (SMD = 0.17) (Begemann et al., 2020). In a meta-analysis investigating rTMS for motor recovery in Parkinson’s disease, HF-rTMS showed a significant effect in enhancing motor function (SMD = 0.48) relative to LF-rTMS in subgroup analysis (Yang et al., 2018a, b, c). It is not surprising to see mixed evidence in clinical populations due to diverse pathological and neurobiological substrates as well as heterogeneity between studies with different rTMS stimulus parameters and study methodologies. For these reasons, research in healthy populations have benefits for the investigation of potential moderating effects of different stimulation parameters and other study factors.

The small sized effects of offline HF-rTMS we currently observed in healthy participants could possibly be due to target-function misalignment or the limited utility of rTMS. In our current meta-analyses, most cognitive functions are aligned with prefrontal regions, leading to the majority of studies (accuracy: 82.6%, RT: 90.9%) targeting frontal regions, predominately the DLPFC. While the DLPFC has previously been identified to play a role in subserving multiple cognitive functions, including those involving attention (Hart et al., 2012), executive functioning (Niendam et al., 2012), memory and learning (Wesley and Bickel, 2014) or perception (Devoto et al., 2018), other cortical targets may be more relevant for modulation of specific cognitive domains (e.g., temporal lobe for memory). It is important to note, however, that HF-rTMS neuromodulatory effects are not limited to the targeted site, with downstream effects previously observed in both functionally and non-functionally or structurally connected regions and networks (Fox et al., 2012). This could also explain the overall small sized non-specific cognitive enhancing effects observed across multiple domains. Alternatively, small sized effects might suggest the possibility that offline HF-rTMS is not a strong tool to enhance cognition; however, a caveat to this is limitations to the current analysis approach which involved accounting for heterogeneity with different outcome measures, stimulation parameters and study designs. Relatively high heterogeneity (I^2^ > 70%) was demonstrated across overall and domain-oriented analyses in the current study. Future empirical studies or larger meta-analyses are required to examine the cognitive effects of offline HF-rTMS targeting other brain regions outside of the frontal cortex and probe the specificity and moderators of these cognitive effects, for example, through the inclusion of comparator targets.

Offline inhibitory cTBS did not disrupt or improve overall cognitive functioning; interestingly, enhancement of the motor domain (k = 3, p = 0.07) and disruption of the attention domain (k = 4, p = 0.07) reached marginal significance with limited studies in accuracy. Mixed findings of cTBS were also reported in a systematic review which assessed the cognitive effects of cTBS given to the DLPFC; cTBS administered to the LDLPFC caused poorer executive function, working memory and cognitive control though improved planning and decision-making (Ngetich et al., 2020). In a meta-analysis which examined the effects of prefrontal TBS, cTBS impaired executive functioning including working memory, inhibition, attentional control, verbal fluency, task-shifting and other complex executive abilities (Lowe et al., 2018). It is generally accepted that cTBS is an inhibitory form of HF-rTMS that induces synaptic suppression and disrupts cognitive functioning (Chung et al., 2016; Huang et al., 2005). Nonetheless, cognitive enhancement might be produced via addition-by-subtraction, namely disruption of cognitive processing within the targeted cortical region which in turn can reorganise a temporary network and enhance compensatory cognitive processes (Luber & Lisanby, 2014). Future research is required to examine the mechanism of cTBS and optimal parameters (e.g., stimulation sites) for modulating cognition.

Pulse frequency is considered one of the critical parameters for moderating the neuromodulatory effects of offline rTMS. Our results supported that iTBS was not inferior to protocols with 10 Hz or greater frequencies for improving accuracy. In two randomised trials which compared the efficacy of iTBS and 10 Hz rTMS in depressive disorders, iTBS demonstrated an equivalent treatment effect to traditional 10 Hz rTMS (Blumberger et al., 2018; Bulteau et al., 2022). Additionally, protocols with pulse frequency greater than 10 Hz all presented positive effect sizes, suggesting higher pulse frequency produces stronger cognitive enhancement. This was also supported by a meta-analysis of online rTMS, revealing 10 Hz stimulation worsened performance (i.e., reduced accuracy or slower reaction times on cognitive tasks across different domains) relative to control stimulation, and the cognitive effect of online 20 Hz rTMS was relatively stronger to 10 Hz protocols for collapsed cognitive domains (Beynel et al., 2019). Higher pulse frequencies may become a promising parameter to manipulate for improving the cognitive efficacy of HF-rTMS in healthy cohorts as well as in neuropsychiatric disorders.

Exploratory subgroup analyses on frequency also found that offline 10 Hz rTMS was relatively greater to other forms of HF-rTMS for improving reaction times. This finding was consistent with several 10 Hz rTMS studies which examined cognitive effects in healthy samples. For example, Vanderhasselt and colleagues conducted a series of studies examining the effects of 10 Hz rTMS on cognitive control and only found significant results for reaction times (Vanderhasselt et al., 2006, 2007, 2010). Cognitive improvement following 10 Hz rTMS has also been found effective in psychiatric disorders, such as depression (O'Connor et al., 2005) and schizophrenia (Guse et al., 2013). However, in the current analysis, most studies (45.5%) investigated pulse frequencies at 10 Hz, which therefore limited the ability to determine the relative effects of other frequencies. Future research is therefore required to verify whether 10 Hz is the optimal stimulus frequency for modulating reaction times with offline HF rTMS.

Although the exploratory analysis for the targeting method did not attain significance for accuracy, an inspection of the effect sizes suggested numerically larger effect sizes for MRI-guided neuronavigation compared to other targeting methods. However, we did not find results favouring fMRI-guided neuronavigation, possibly because only limited studies (N = 5) used this method in the current analyses. Neuronavigation approaches are associated with stronger cognitive effects of HF-rTMS, which corroborates evidence from previous observations by Beynel et al. (2019) who reported that individualised fMRI guided targets were associated with greater cognitive effects with online rTMS. It was interesting to find that use of the 10–20 EEG system was a relatively greater localisation approach compared to other targeting methods for reaction times. This result reflected those of Hebel et al. (2021) who showed that 10–20 EEG system guided iTBS was not an inferior localisation approach relative to MRI-based neuronavigation for patients with depression. Future studies are required to confirm whether the neuronavigated targeting approach or the 10–20 EEG system is the optimal targeting method for enhancing cognition with offline HF-rTMS.

We additionally found evidence in support of a moderating effect of different control conditions on the cognitive effects of offline HF-rTMS for both accuracy and reaction times. Specifically, use of a sham coil or equivalent (e.g., specialised sham coil, using spacer, setting stimulation intensity to 0% or combined approaches) demonstrated a relatively larger effect for moderating accuracy after HF-rTMS, suggesting sham coils are better for showing benefits of HF-rTMS for cognitive outcomes. It is possible that other control methods may induce unintentional cognitive effects due to HF-rTMS inducing activation in downstream cortical or subcortical brain regions. Surprisingly, we did not find evidence supporting greater effects from using a sham coil for reaction time, though use of an angle rotation was associated with greater effects compared to the other control approaches. However, for this analysis 65.9% of studies adopted use of an angle rotation (i.e., coil tilted at 45° or 90°), and as such there was limited statistical power to assess the influence of alternative methods. Further research is needed to confirm whether use of sham coil or an angle rotation may play different roles in moderating the cognitive effects of offline HF-rTMS.

Even though the subgroup analyses of the effect of number of sessions was not statistically significant, observation of the effect sizes suggested that multiple sessions may be associated with relatively larger effects for both accuracy [0.04 (single session) vs. 0.15 (multiple sessions)] and reaction times [-0.07 (single session) vs. -0.19 (multiple sessions)]. Similarly, greater cognitive effects with multiple sessions have been observed in neuropsychiatric populations, including depression (Martin et al., 2017; Schulze et al., 2018), stroke (Kim et al., 2015) and Parkinson's disease (Jiang et al., 2020). Future studies are required to verify whether utilising multiple sessions of offline HF-rTMS may induce greater cognitive enhancing effects in healthy cohorts.

We observed substantial heterogeneity in meta-analyses across cognitive domains. It is possible that methodological and sample differences between studies may account for this finding. Potential moderators of heterogeneity could include diverse cognitive tasks and limited sample sizes. Data analysis from diverse cognitive tasks even within the same cognitive domain is potentially an issue as underlying cognitive processes may be distinct. For example, task-specific cognitive effects of offline rTMS administered to the DLPFC were observed in patients with depression (Martin et al., 2017) and cognitive effects of non-invasive brain stimulation in healthy cohorts at task level were also reported in a recent meta-analysis (de Boer et al., 2021). The current sample included 80 different cognitive tasks grouped across six domains. The executive functioning domain included tasks that assessed abilities including updating ability, shifting ability and inhibition ability consistent with commonly accepted definitions (Miyake & Friedman, 2012). Aggregating effects from different tasks thus likely contributes to heterogeneity. In addition, the majority of included studies had limited sample sizes (N < 30) with small to moderate effect sizes, which results in greater variability for reported effects. Future research would benefit from including larger sample sizes and examination of inter-individual factors (e.g., physiological markers) potentially related to variability in responsivity.

### Strengths and Limitations

Strengths of the current review included restricting analyses to randomised controlled studies, the categorisation of cognitive tasks into domains of cognitive function to investigate domain specific effects, and the examination of potential moderating factors. Notwithstanding, there were several limitations which deserve consideration: (1) data were included from studies which used diverse cognitive tasks, which may have limited ability to observe effects for more specific cognitive processes, (2) it is possible that lack of cognitive effects may be due to insufficient target-function alignment, (3) the majority of studies targeted the frontal lobe which could limit potential generalisation of current findings to other brain regions, (4) limited studies were available for some cognitive domains and exploratory subgroup analyses, (5) included studies were restricted to healthy populations which could limit the magnitude of effect sizes due to the potential for ceiling effects, (6) averaging effect sizes across multiple outcome measures and cognitive tasks in the same domain may lose specificity for true effect sizes for specific functions within a domain or task (7) analysed studies were restricted to those written in the English language.

## Conclusions

Overall, the current systematic review and meta-analysis found evidence for small sized cognitive enhancing effects of offline HF-rTMS for both accuracy and reaction time contributed by most cognitive domains. Significant accuracy improvement was only observed for the executive functioning domain and significant improvement in reaction times was limited to the executive function and motor domains. Despite its exploratory nature, this study offers some insight into moderators of cognitive enhancement with offline HF-rTMS. Our results suggested that cognitive effects may depend on stimulation pulse frequency, targeting methods and control comparators. Taken together, further controlled studies are required to fully ascertain the specificity of cognitive effects with offline HF-rTMS and the relative effects of different stimulation parameters for improving cognitive functioning.

### Supplementary Information

Below is the link to the electronic supplementary material.

Supplementary file1 (DOCX 2935 KB)

## Data Availability

Data and R scripts used for meta-analyses are available at the following link: https://github.com/EchoXu9/Offline_HF-rTMS_Cognition
