# Supplementary material for: Cognitive Effects Following Offline High-Frequency Repetitive Transcranial Magnetic Stimulation (HF-rTMS) in Healthy Populations: A Systematic Review and Meta-Analysis
Source: Neuropsychol Rev. 2023 Mar 1;34(1):250–76. doi: 10.1007/s11065-023-09580-9 (PMC10920443; doi:10.1007/s11065-023-09580-9)
Supplement: Supplementary file 1 — Supplementary file1 (DOCX 2935 KB) [file 11065_2023_9580_MOESM1_ESM.docx]

**Supplementary Materials**

Cognitive enhancing effects following offline high-frequency repetitive transcranial magnetic stimulation (HF-rTMS) in healthy populations: a systematic review and meta-analysis

Mei Xu^1^, Stevan Nikolin^1, 2^, Nisal Samaratunga^1^, Esther Jia Hui Chow^1^, Colleen K. Loo^1, 2, 3^, Donel M. Martin^1,2*^

^1^School of Psychiatry, Faculty of Medicine, University of New South Wales, Sydney, Australia

^2^Black Dog Institute, Sydney, Australia

^3^The George Institute for Global Health

**Correspondence**:

Donel M. Martin

donel.martin@unsw.edu.au

**Table S1**

Equations of effect sizes computation

|  | **Parallel controlled trials** | **Cross-over controlled trials** |
| --- | --- | --- |
| **SD_A,change_** | $\sqrt{{SD}_{A,pre}^{2}+{SD}_{A,post}^{2}-(2\times r_{pre\&post}\times{SD}_{A,pre}\times{SD}_{A,post})}$ **(1)** | |
| **SD_c,change_** | $\sqrt{{SD}_{C,pre}^{2}+{SD}_{C,post}^{2}-(2\times r_{pre\&post}\times{SD}_{C,pre}\times{SD}_{C,post})}$ **(2)** | |
| **SMD** | $SMD = \frac{M_{A,change}-M_{C,change}}{\sqrt{\frac{\left( n_{A}-1 \right){SD}_{A,change}^{2}-\left( n_{C}-1 \right){SD}_{C,change}^{2}}{n_{A}+n_{C}-2}}}$ **(3)**  ${V_{d}}^{2} = \frac{n_{A}+ n_{C}}{n_{A}\times n_{C}} + \frac{{SMD}^{2}}{2(n_{A}+ n_{C})}$ **(4)** | $SMD= \frac{M_{A,change}-M_{C,change}}{\frac{{SD}_{diff}}{\sqrt{2(1-r_{A\&C})}}}$ **(5)**  ${SD}_{diff}=\sqrt{{SD}_{A,change}^{2}+{SD}_{C,change}^{2}-\left( 2\times r_{A\&C}\times{SD}_{A,change}\times{SD}_{C, change} \right)}$  ${V_{d}}^{2} =\left( \frac{1}{n_{\mathrm{pairs}}} + \frac{\mathrm{SMD}^{2}}{2n_{\mathrm{pairs}}} \right)\times2(1-r_{A\&C})$ **(6)** |
| **Hedges’ g** | $Hedges^{'}g=SMD \times J$ **(7)**  $V_{g} = V_{d} \times J^{2}$ **(8)**  $J=1 -\frac{3}{4df -1}, df=n_{A}+n_{C}-2$ | $Hedges^{'}g=SMD \times J$ **(9)**  $V_{g} = V_{d} \times J^{2}$ **(10)**  $J=1 -\frac{3}{4df -1}, df=n_{pairs}-1$ |

Note: ${SD}_{A,change}$ : standard deviation of cognitive change scores for active group; ${SD}_{C,change}$ : standard deviation of cognitive change scores for control group; ${SD}_{A,pre}$ : standard deviation of pre-stimulation cognitive outcomes for active group; ${SD}_{A,post}$: standard deviation of post-stimulation cognitive outcomes for active group; $r_{pre\&post}$: correlation between pre- and post-stimulation; ${SD}_{C,pre}$ : standard deviation of pre-stimulation cognitive outcomes for control group; ${SD}_{C,post}$ : standard deviation of post-stimulation cognitive outcomes for control group; SMD: standardized mean difference; $M_{A,change}$ : mean change score of active group; $M_{C,change}$ : mean change score of control group; $n_{A}$ : sample size of active group; $n_{C}$ : sample size of control group; $V_{d}$: Variance of SMD; $r_{A\&C}$ : correlation between active and control condition; $n_{\mathrm{pairs}}$ : sample size of cross-over trials.

**Table S2**

Cognitive variables selected in the analyses

| **Cognitive domain** | **Cognitive tasks** | **k** |
| --- | --- | --- |
| Attention | 0-back  1-back  Free visual exploration task  Exogenous cueing task (ECT)  Stroop task  Oddball paradigm  TAP-alertness  TAP-divided attention  Reaction task  Multiple object tracking (MOT)  Rapid visual information processing task (RVIP)  Continuous performance task (CPT)  Self-matching task  Trial making test (TMT)-Part A  Reaction time tasks  Digit span task (DSP)-Forward | 3  4  1  1  7  1  1  1  1  1  1  1  1  1  1  1 |
| Motor | Fitts’ paradigm  Motor sequency experiment  Sensory discrimination measurement  TULIA  Postural imitation test  Nine-hole peg test (NHPT)  Purdue pegboard test (PPT)  Minnesota dexterity task (MDT)  Two-ball rotation task  Continuous tracking task | 1  1  1  1  1  1  1  1  1  1 |
| Memory | Pattern recognition memory task (PRM)  Spatial recognition memory task (SRM)  Serial reaction time task (SRTT)  Memory test  Aiming and memory task  Episodic memory task  Face-cued word recall testing  Associative memory task | 1  1  1  2  1  1  1  1 |
| Language | Sentence repetition task | 1 |
| Perception | Conscious detection task  Filling-in paradigm  Emotion discrimination task  Identity discrimination task  Vocal identity discrimination task  Order and quantity tasks  Achromatic motion discrimination task  Chromatic motion discrimination task  Chromatic detection task  Achromatic detection task  Conjunction search task  Feature search task  Perceptual task  Line bisection (LB)  Landmark tasks (LM)  Mental rotation task | 1  1  1  1  1  1  1  1  1  1  1  1  1  1  1  1 |
| Executive function | Digit span task (DSP)-Backward  Spatial 2‐back task (S2B)  Delayed match‐to‐sample task (DMS)  Spatial span task (SSP)  Stockings of Cambridge task (SOC)  2-back  3-back  Stroop task  Negative affective priming task (NAP)  Colour task  Orientation task  Dual task  TAP-go/no-go  TAP-working memory  TAP-flexibility  Verbal fluency tasks  Ruff figural fluency test  Adjusting amount task (AAT)  Information sampling task (IST)  Stop signal task (SST)  Change detection task  Self-matching task  Raven’s progressive matrices  Stop-switching task  Trial making test (TMT)-Part B  Game of dice task (GDT)  Risky gains task (RGT)  Agency task  Spatial working memory task | 2  1  1  1  1  5  5  8  1  1  1  1  2  1  1  1  1  1  2  2  1  1  1  1  1  1  1  1  1 |


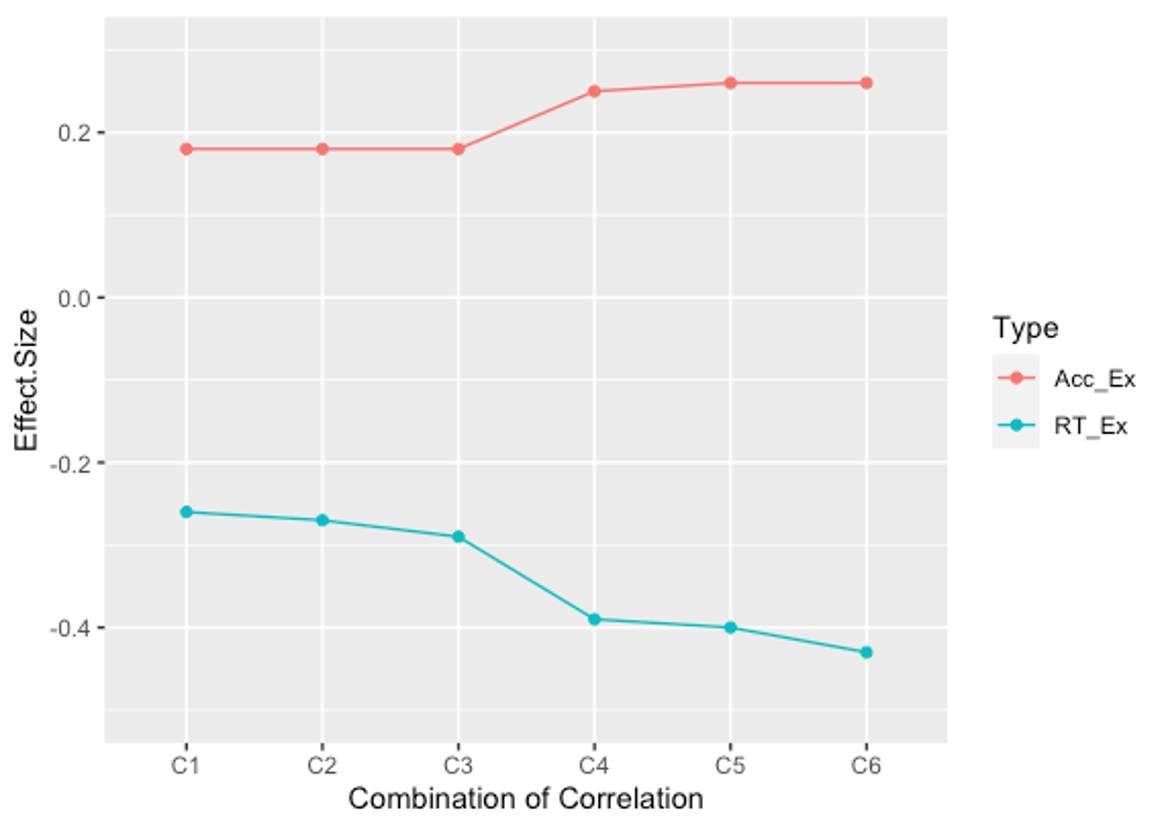


**Fig. S1** Hedge’s g values with six correlation coefficient values.

*Note:* C1: r_pre-post_= 0, r_active-control_=0; C1: r_pre-post_= 0, r_active-control_=0.5; C1: r_pre-post_= 0, r_active-control_=0.8; C1: r_pre-post_= 0.5, r_active-control_=0; C1: r_pre-post_= 0.5, r_active-control_=0.5; C1: r_pre-post_= 0.5, r_active-control_=0.8; Acc_Ex: overall effect size of excitatory studies for accuracy; RT_Ex: overall effect size of excitatory studies for reaction time; This sensitive analysis calculated the overall effect sizes from 45 studies.


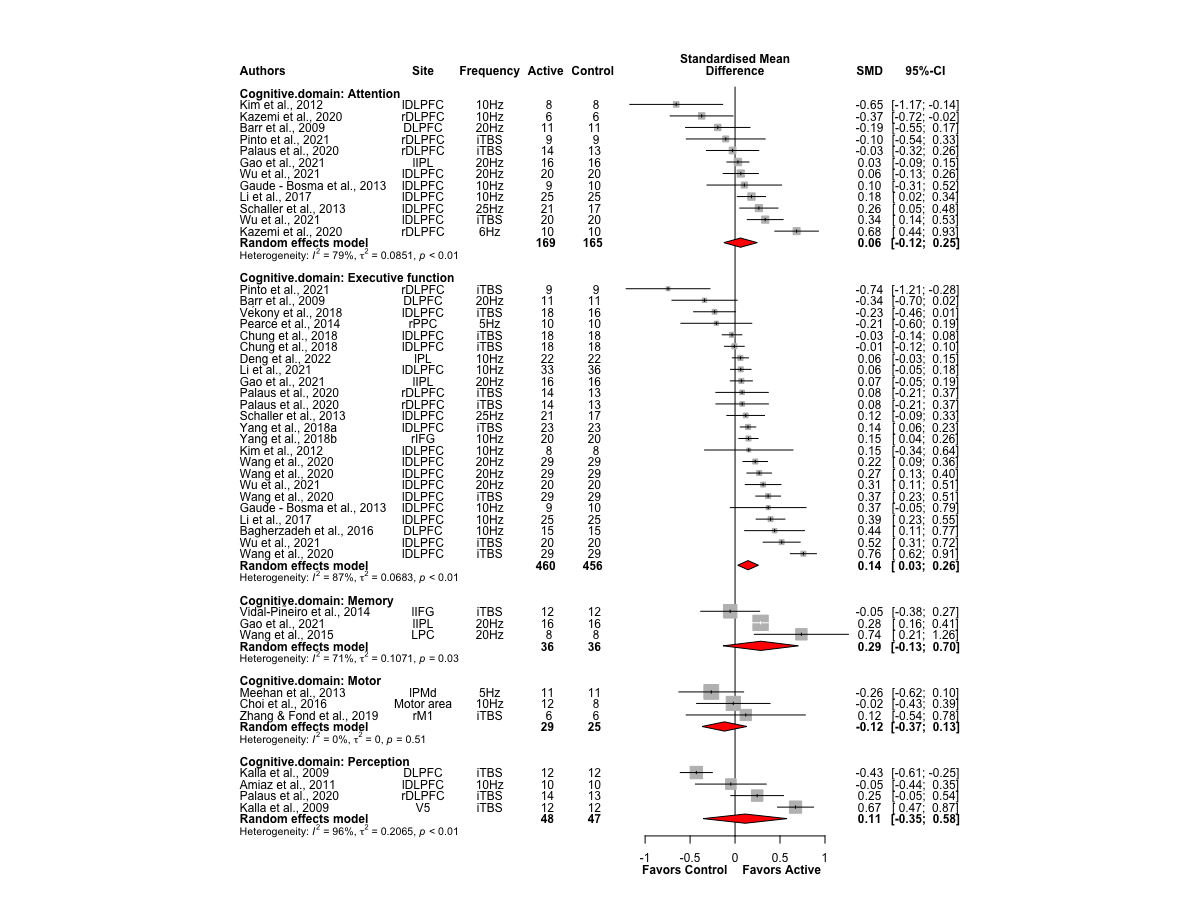


**Fig. S2** Forest plots of effects of excitatory HF-rTMS across cognitive domains for accuracy

*Note*: l: Left; r: Right; b: Bilateral; DLPFC: Dorsolateral prefrontal cortex; IPL: Inferior parietal lobule; PPC: Posterior parietal cortex; PL: Parietal lobe; IFG: Inferior Frontal Gyrus; PC: Parietal cortex; PMd: dorsal premotor cortex; M1: Primary motor cortex; V5: Visual cortex


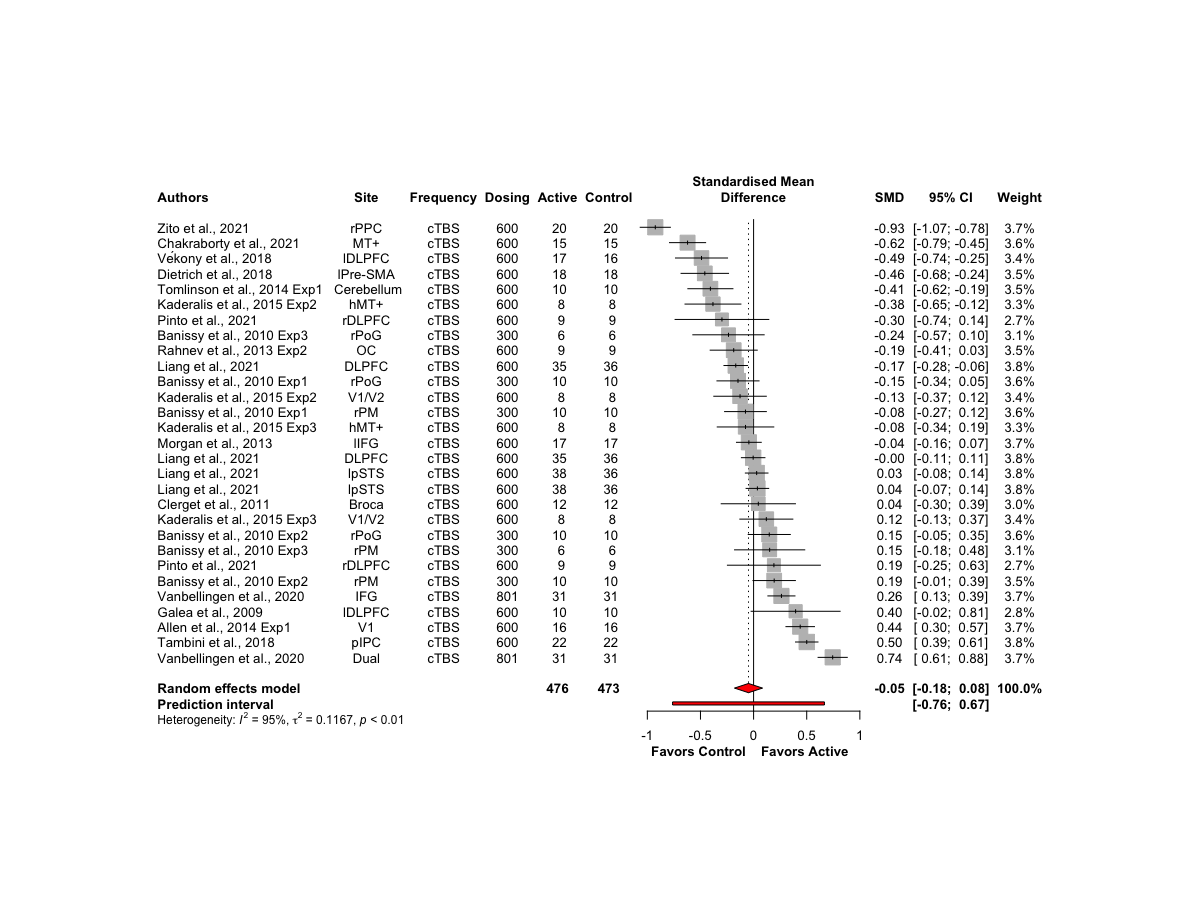


**Fig. S3** Forest plots of effects of inhibitory HF-rTMS for accuracy

*Note:* Dosing represents the total pulses per session; l: Left; r: Right; b: Bilateral; PPC: Posterior parietal cortex; MT+: middle temporal area and medial superior temporal area; DLPFC: Dorsolateral prefrontal cortex; SMA: Supplementary motor area; PoG: Postcentral gyrus; OC: Occipital cortex; V1: Primary visual cortex; PM: Lateral premotor cortex; hMT+: The point that elicited the strongest moving phosphene; IFG: Inferior frontal gyrus; pSTS: Posterior superior temporal sulcus; pIPC: Posterior inferior parietal cortex; Dual: targeted at dual sites (Left IFG and right IPL: Inferior parietal lobule)


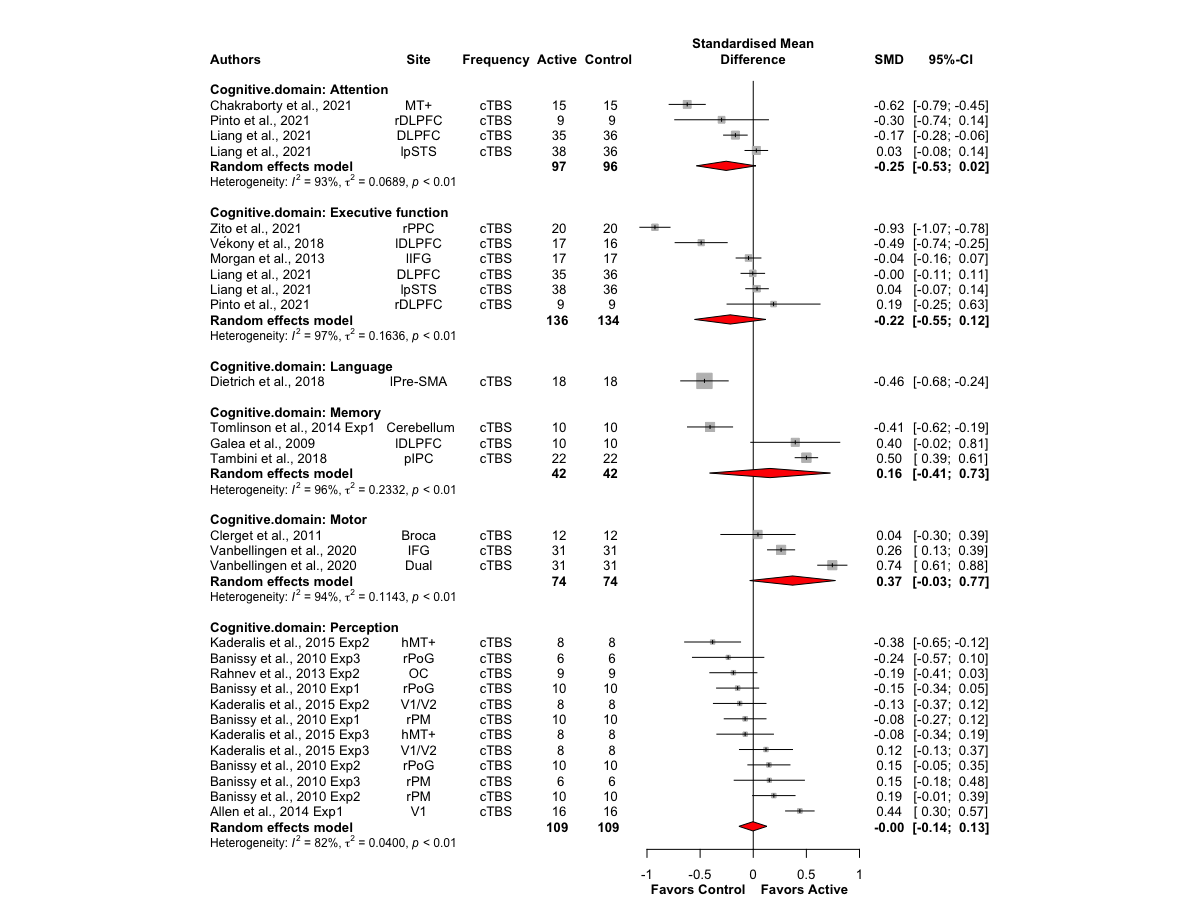


**Fig. S4** Forest plots of effects of inhibitory HF-rTMS across cognitive domains for accuracy

*Note*: l: Left; r: Right; b: Bilateral; MT+: middle temporal area and medial superior temporal area; DLPFC: Dorsolateral prefrontal cortex; pSTS: Posterior superior temporal sulcus; PPC: Posterior parietal cortex; IFG: Inferior frontal gyrus; SMA: Supplementary motor area; pIPC: Posterior inferior parietal cortex; Dual: targeted at dual sites (Left IFG and right IPL: Inferior parietal lobule); hMT+: The point that elicited the strongest moving phosphene; PoG: Postcentral gyrus; OC: Occipital cortex; V1: Primary visual cortex; PM: Lateral premotor cortex


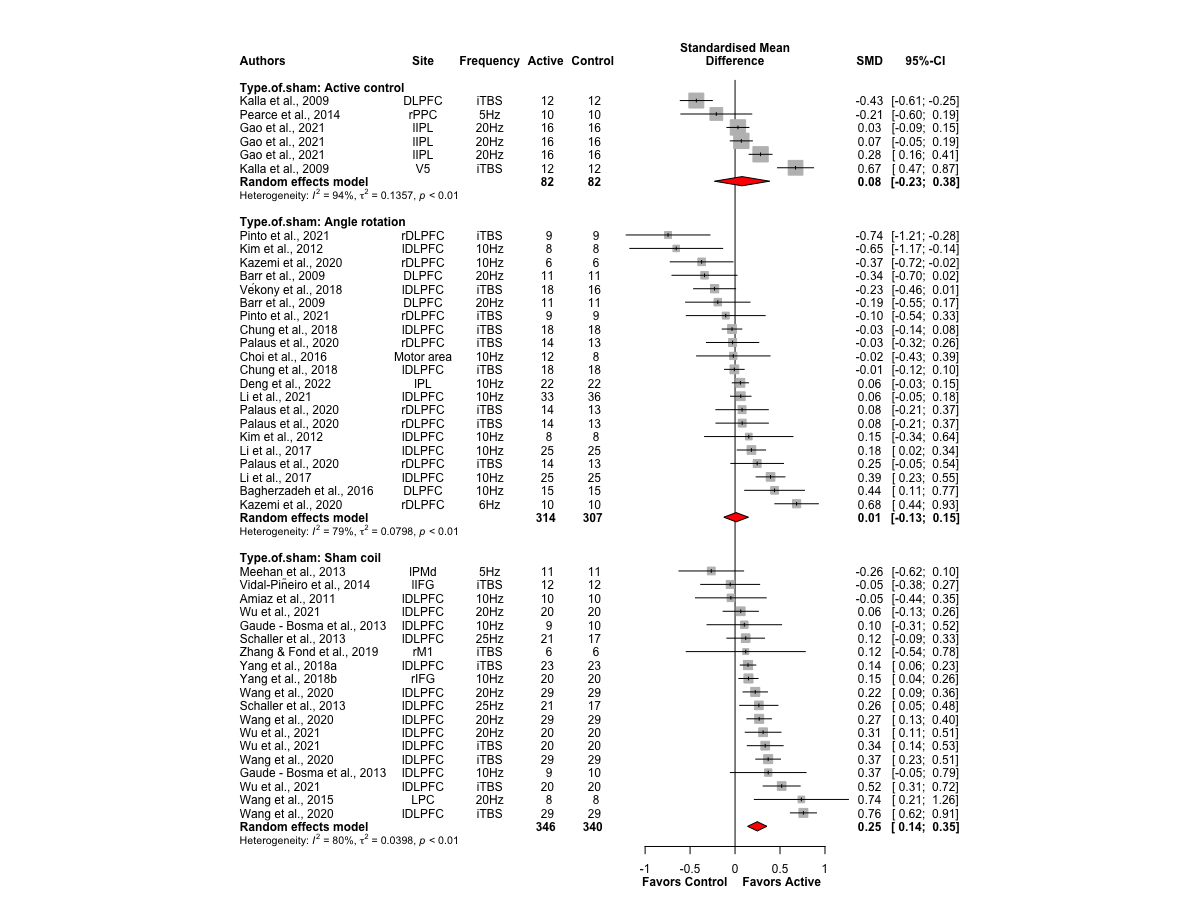


**Fig. S5** Forest plots for effects of excitatory HF-rTMS on control methods for accuracy

*Note*: l: Left; r: Right; b: Bilateral; DLPFC: Dorsolateral prefrontal cortex; PPC: Posterior parietal cortex; IPL: Inferior parietal lobule; V5: Visual cortex; PL: Parietal lobe; PMd: dorsal premotor cortex; IFG: Inferior Frontal Gyrus; M1: Primary motor cortex; PC: Parietal cortex


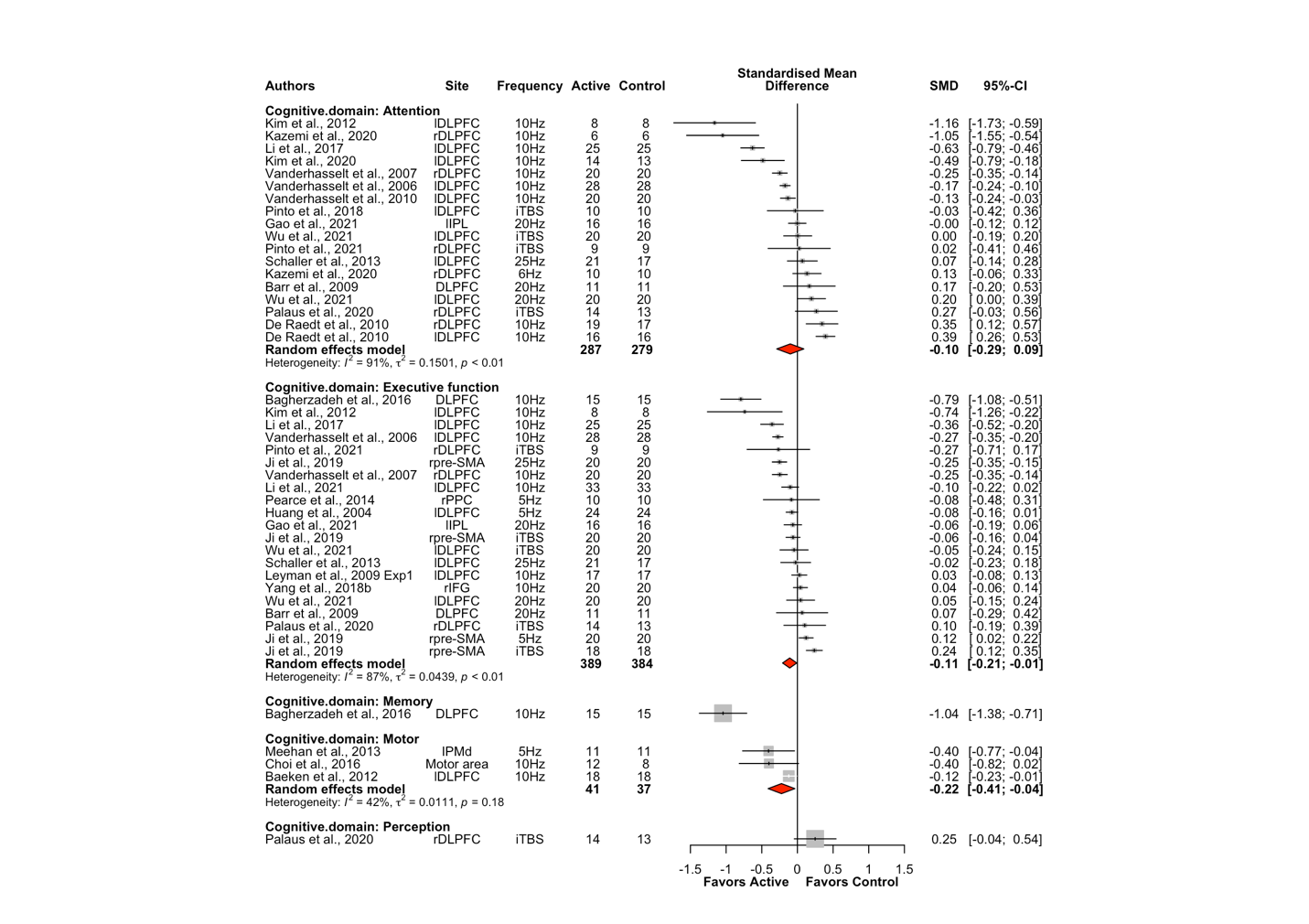


**Fig. S6** Forest plots of effects of excitatory HF-rTMS across cognitive domains for reaction time

*Note*: l: Left; r: Right; b: Bilateral; DLPFC: Dorsolateral prefrontal cortex; IPL: Inferior parietal lobule; SMA: Supplementary motor area; PPC: Posterior parietal cortex; IFG: Inferior frontal gyrus; PMd: dorsal premotor cortex


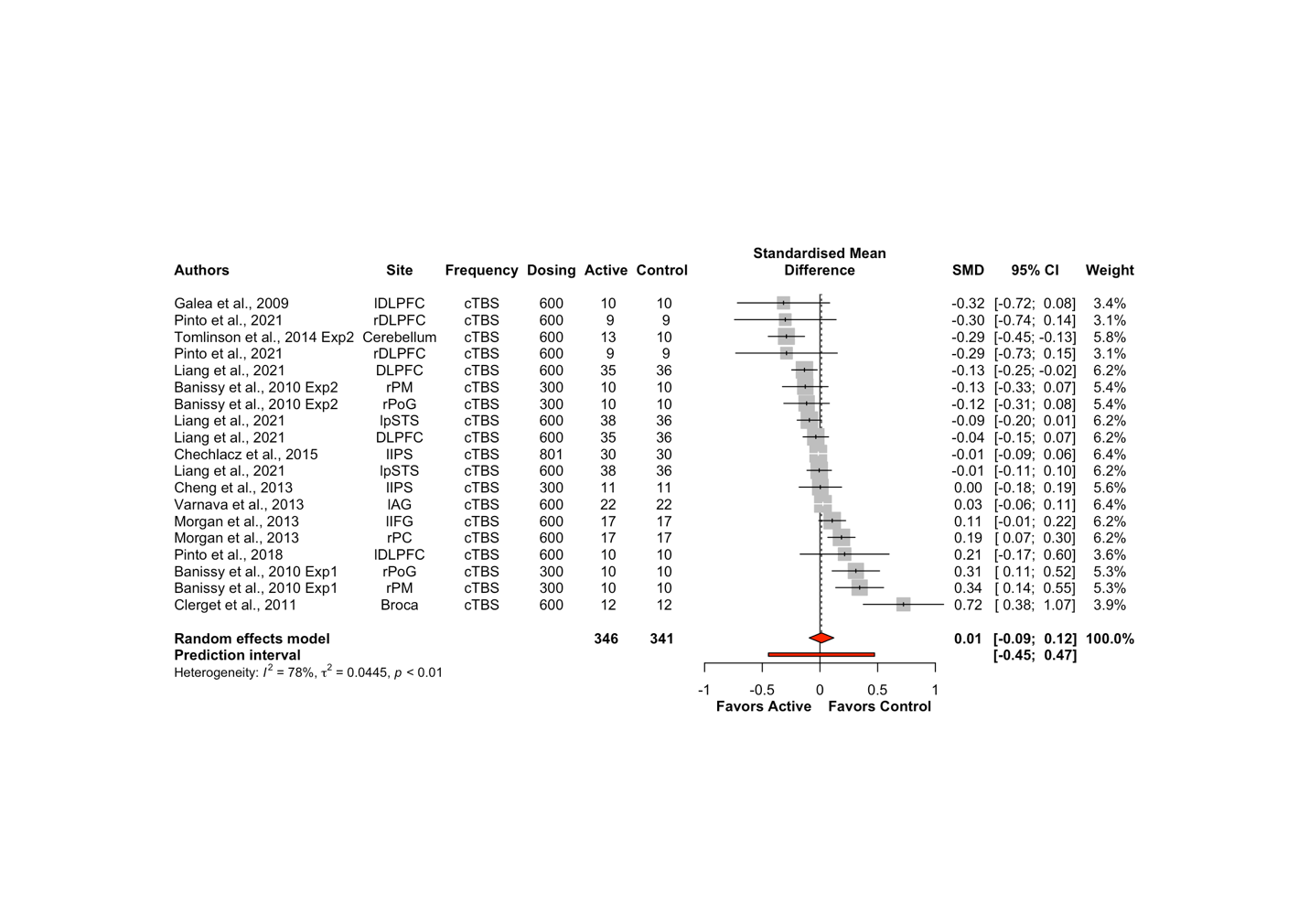


**Fig. S7** Forest plots of effects of inhibitory HF-rTMS for reaction time

*Note:* Dosing represents the total pulses per session; l: Left; r: Right; b: Bilateral; DLPFC: Dorsolateral prefrontal cortex; PM: Lateral premotor cortex; PoG: Postcentral gyrus; pSTS: Posterior superior temporal sulcus; IPS: Intraparietal sulcus; AG: Angular gyrus; IFG: Inferior frontal gyrus; PC: Parietal cortex


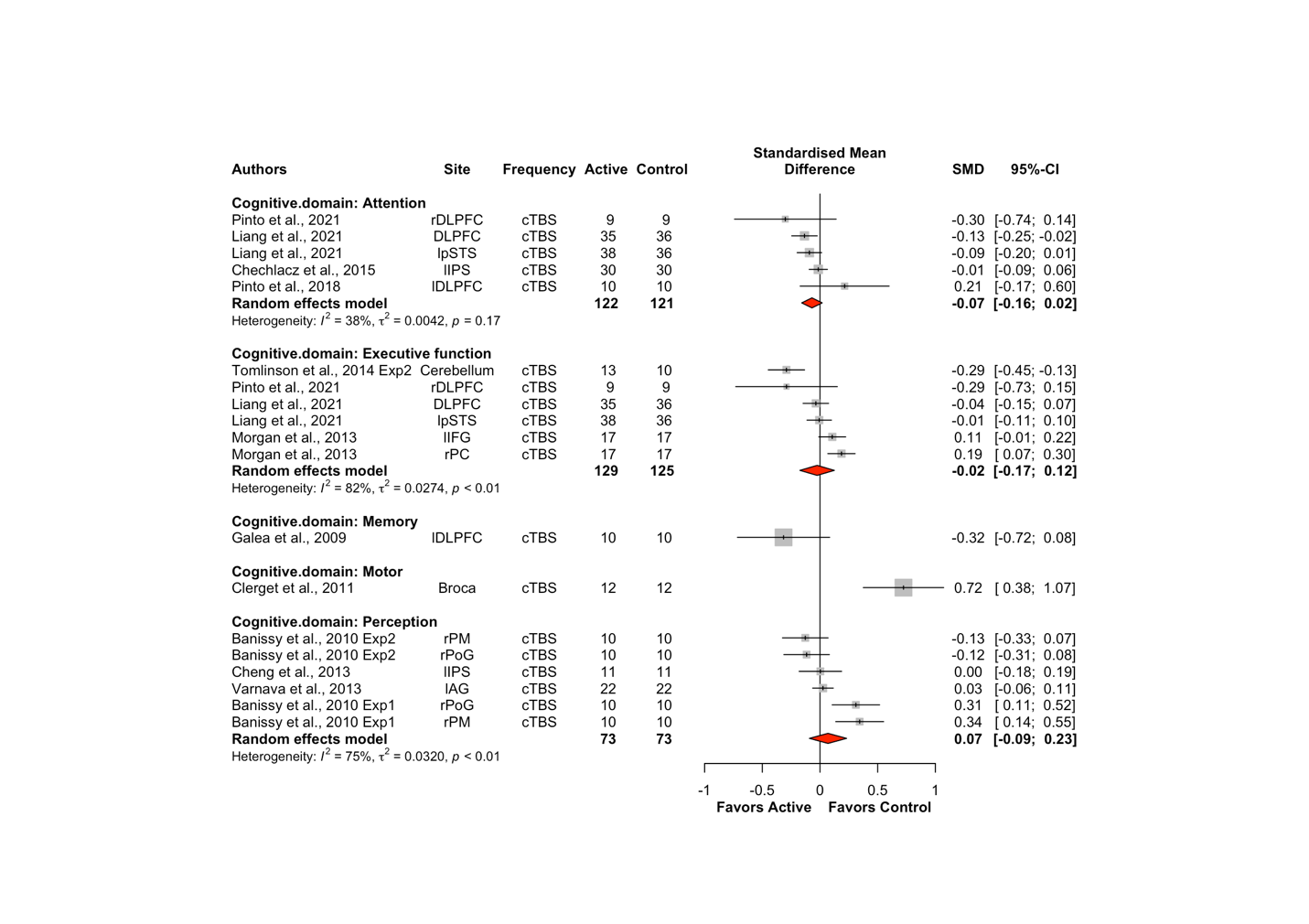


**Fig. S8** Forest plots of effects of inhibitory HF-rTMS across cognitive domains for reaction time

*Note*: l: Left; r: Right; DLPFC: Dorsolateral prefrontal cortex; pSTS: Posterior superior temporal sulcus; IPS: Intraparietal sulcus; IFG: Inferior frontal gyrus; PC: Parietal cortex; PM: Lateral premotor cortex; PoG: Postcentral gyrus; AG: Angular gyrus.


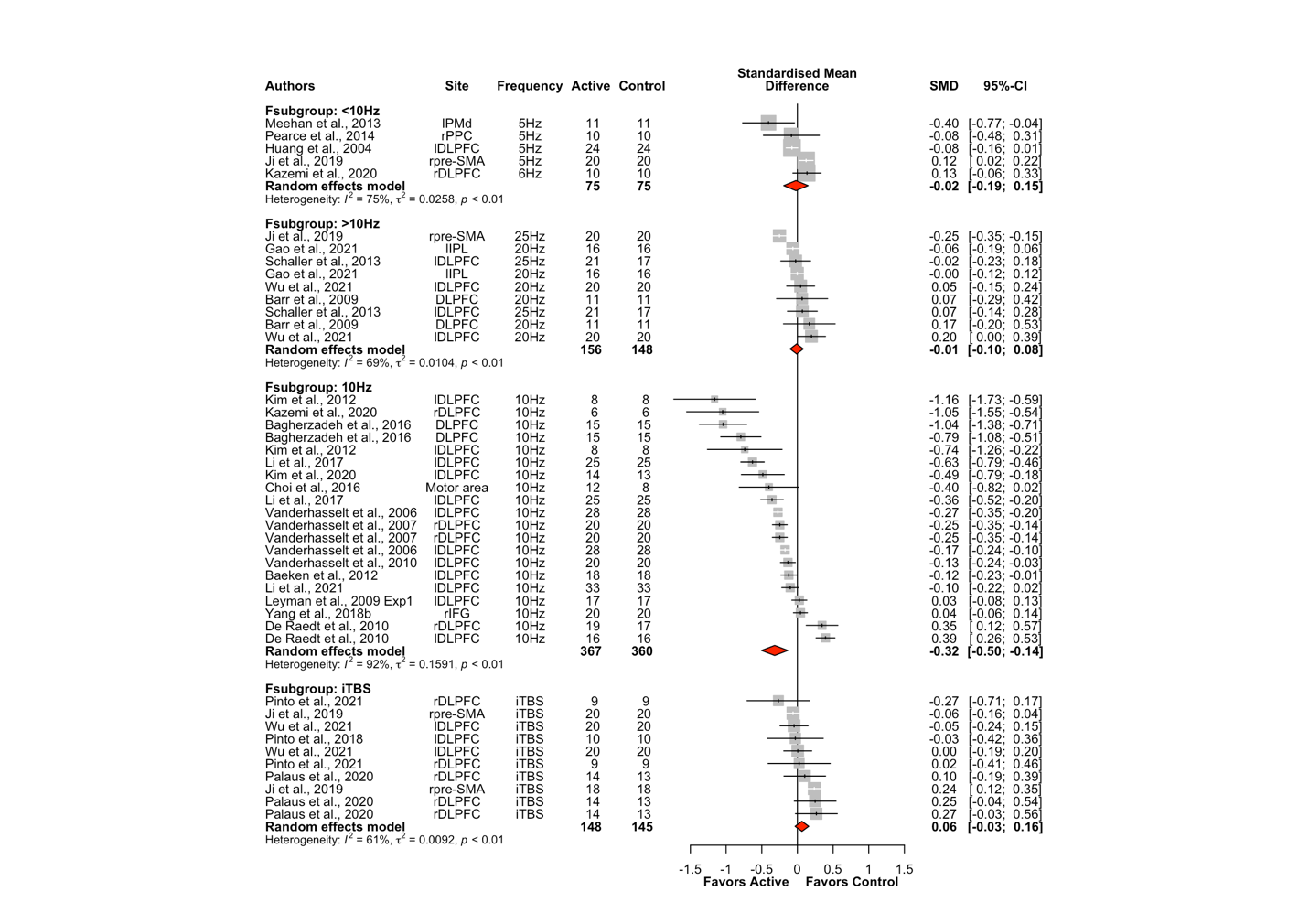


**Fig. S9** Forest plots for effects of excitatory HF-rTMS on frequency for reaction time

*Note*: l: Left; r: Right; b: Bilateral; PMd: dorsal premotor cortex; PPC: Posterior parietal cortex; DLPFC: Dorsolateral prefrontal cortex; SMA: Supplementary motor area; IPL: Inferior parietal lobule; IFG: Inferior frontal gyrus


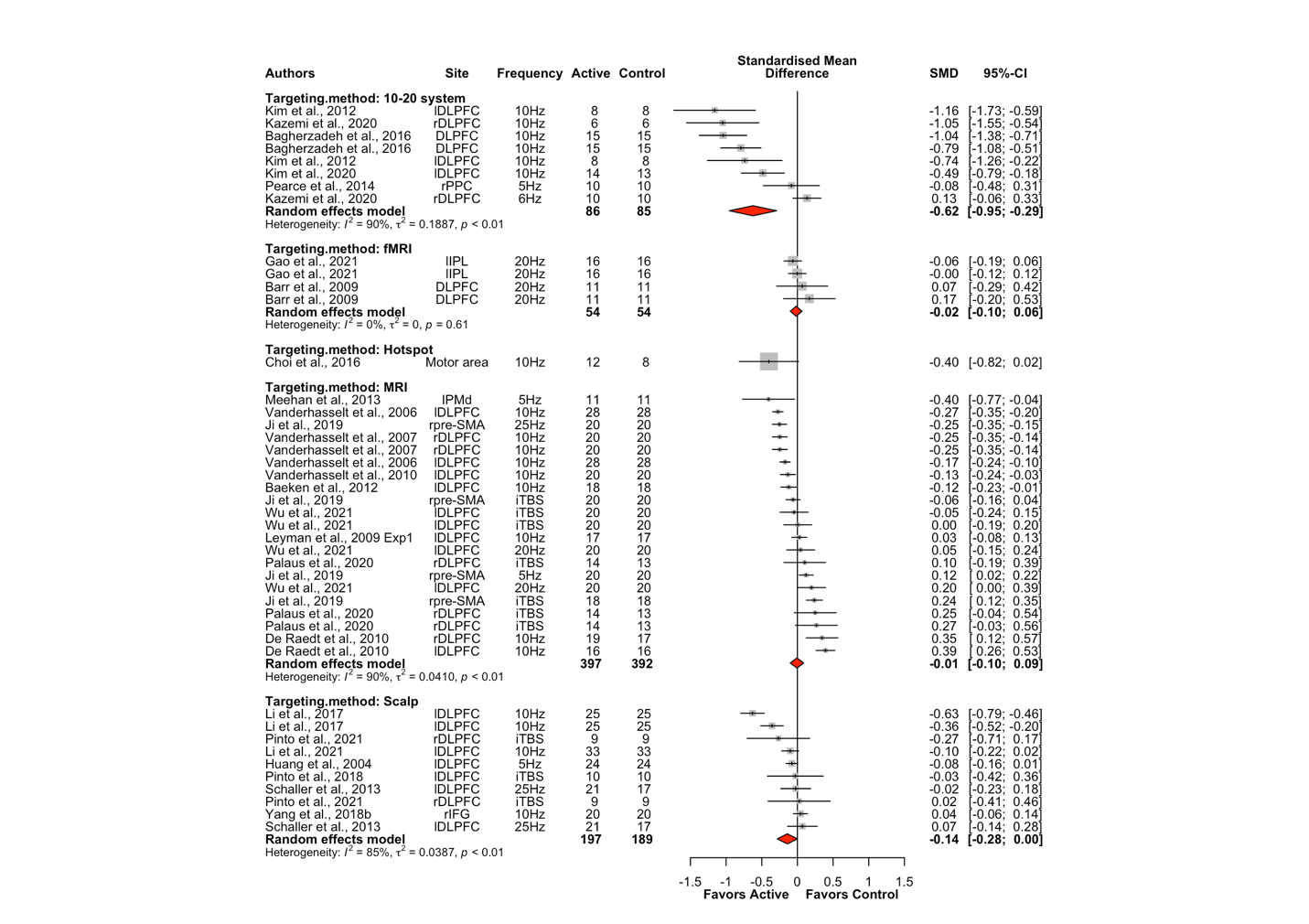


**Fig. S10** Forest plots for effects of excitatory HF-rTMS on targeting methods for reaction time

*Note*: l: Left; r: Right; b: Bilateral; DLPFC: Dorsolateral prefrontal cortex; PPC: Posterior parietal cortex; IPL: Inferior parietal lobule; PMd: dorsal premotor cortex; SMA: Supplementary motor area; IFG: Inferior frontal gyrus


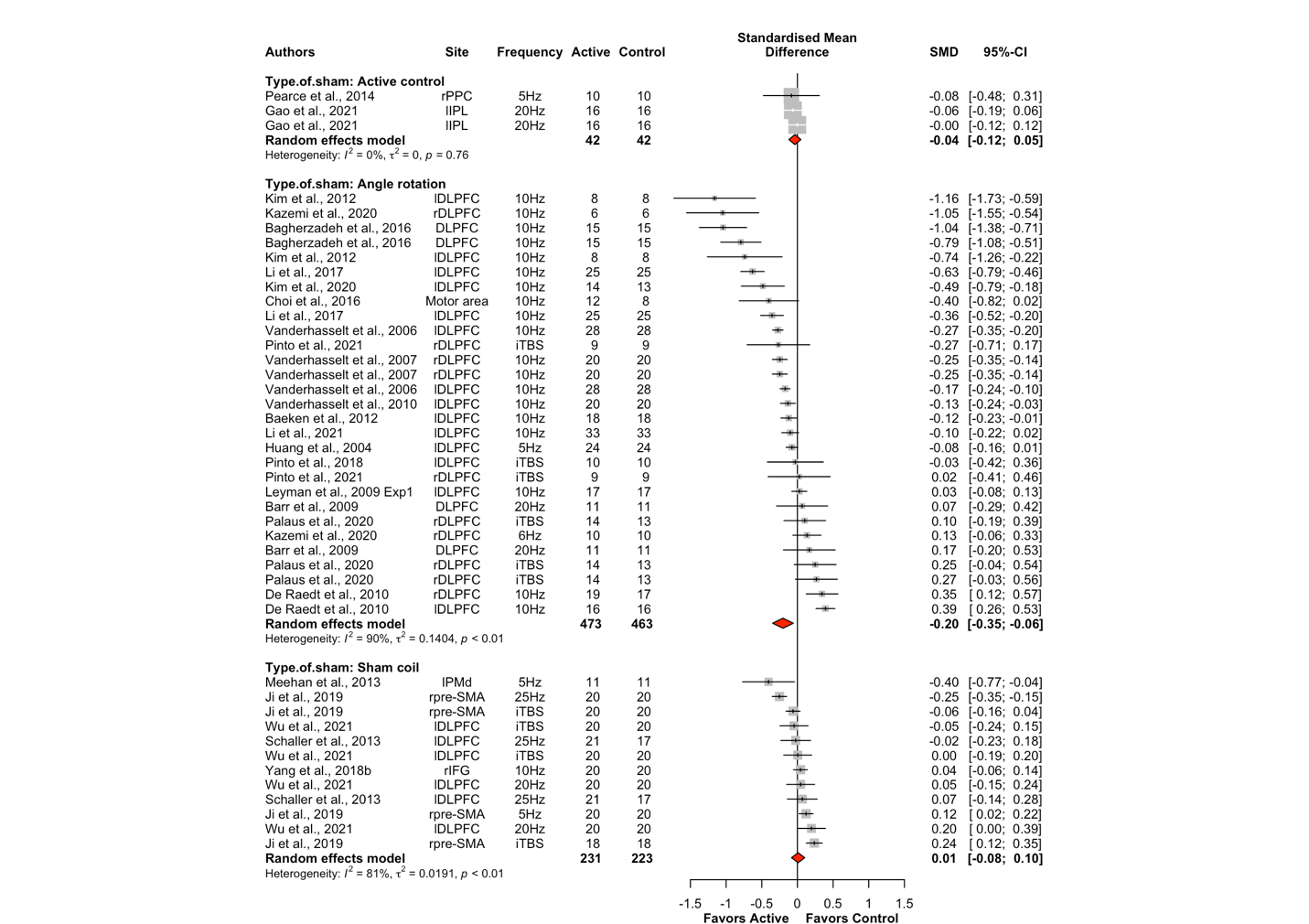


**Fig. S11** Forest plots for effects of excitatory HF-rTMS on control methods for reaction time

*Note*: l: Left; r: Right; b: Bilateral; PPC: Posterior parietal cortex; IPL: Inferior parietal lobule; DLPFC: Dorsolateral prefrontal cortex; PMd: dorsal premotor cortex; SMA: Supplementary motor area; IFG: Inferior frontal gyrus
